# Estimating Sink Parameters of Stochastic Functional-Structural Plant Models Using Organic Series-Continuous and Rhythmic Development

**DOI:** 10.3389/fpls.2018.01688

**Published:** 2018-11-30

**Authors:** Mengzhen Kang, Jing Hua, Xiujuan Wang, Philippe de Reffye, Marc Jaeger, Sélastique Akaffou

**Affiliations:** ^1^The State Key Laboratory of Management and Control for Complex Systems, LIAMA, Institute of Automation, Chinese Academy of Sciences, Beijing, China; ^2^Innovation Center for Parallel Agriculture, Qingdao Academy of Intelligent Industries, Qingdao, China; ^3^Beijing Engineering Research Center of Intelligent Systems and Technology, Beijing, China; ^4^CIRAD, Amap Unit, Univ. Montpellier, CNRS, INRA, IRD, Montpellier, France; ^5^Department of Seeds and Seedlings Production, University Jean Lorougnon Guédé, Daloa, Ivory Coast

**Keywords:** greenlab, inverse method, source-sink parameters, functional-structural plant model, stochastic development, parameter estimation

## Abstract

Functional-structural plant models (FSPMs) generally simulate plant development and growth at the level of individual organs (leaves, flowers, internodes, etc.). Parameters that are not directly measurable, such as the sink strength of organs, can be estimated inversely by fitting the weights of organs along an axis (organic series) with the corresponding model output. To accommodate intracanopy variability among individual plants, stochastic FSPMs have been built by introducing the randomness in plant development; this presents a challenge in comparing model output and experimental data in parameter estimation since the plant axis contains individual organs with different amounts and weights. To achieve model calibration, the interaction between plant development and growth is disentangled by first computing the occurrence probabilities of each potential site of phytomer, as defined in the developmental model (potential structure). On this basis, the mean organic series is computed analytically to fit the organ-level target data. This process is applied for plants with continuous and rhythmic development simulated with different development parameter sets. The results are verified by Monte-Carlo simulation. Calibration tests are performed both *in silico* and on real plants. The analytical organic series are obtained for both continuous and rhythmic cases, and they match well with the results from Monte-Carlo simulation, and vice versa. This fitting process works well for both the simulated and real data sets; thus, the proposed method can solve the source-sink functions of stochastic plant architectures through a simplified approach to plant sampling. This work presents a generic method for estimating the sink parameters of a stochastic FSPM using statistical organ-level data, and it provides a method for sampling stems. The current work breaks a bottleneck in the application of FSPMs to real plants, creating the opportunity for broad applications.

## Introduction

Plant architecture, which is derived from the concept of plant morphology, is the result of endogenous growth processes and exogenous environment conditions (Barthélémy and Caraglio, 2007), hinting that the endogenous growth processes can be inferred from plant architecture and the environment. Functional-structural plant models (FSPMs) aim to represent three-dimensional (3D) plant structure by combining physiological functions (Vos et al., 2010) to create a bridge linking the joint effect of internal growth and the external environment with the visible plant architecture. The state of the art of FSPM and its applications were recently reviewed in the special issue of the FSPMA2016 joint conference (Evers et al., 2018). In recent years, the focus of FSPM has switched from the reconstruction of static 3D plant and canopy architecture for analyzing the effects of plant traits on light capture to the dynamic simulation of plant growth and development determined by the underlying eco-physiological processes (such as photosynthesis and allocation of assimilates).

In contrast to traditional process-based plant models (e.g., TomSim, Heuvelink, 1999) that address carbon production and allocation at the plant level, the spatial scale of FSPMs is generally at the individual phytomer/organ level, which is an important intermediate scale linking the cell level to plant- or field-level studies in systems biology. However, an FSPM cannot be used to draw reliable conclusions without being parameterized. Here, the term “parameterization” refers to the estimation of developmental and functional (source and sink) parameters controlling dynamic growth processes as opposed to the reconstruction of static 3D plant structures using image-based approaches or laser scanning. Some calibrations occur at the submodel level by incorporating measured, empirical values (Garin et al., 2018; Perez et al., 2018; Robert et al., 2018), or by individual parameter estimation (Gu et al., 2018; Perez et al., 2018; Zhu et al., 2018), while some calibrations are performed at the whole-model level (Bongers et al., 2018; Ma et al., 2018; Robert et al., 2018). Among the submodels, photosynthesis modeling, which dates back to the classical work of Farquhar (Farquhar and Roderick, 2003), has been studied extensively at the leaf and canopy levels in recent years with the support of plant 3D structure (Li and Tang, 2017). Currently, a great deal of attention in FSPM is devoted to transport and allocation processes within plants (Evers et al., 2018). Allocation (sink control) is an active area of research that has clearly benefited from the application of an FSPM approach. However, sink function or transportation parameters usually do not have clear physiological meaning, and their values greatly depend on the context of the model. The inverse method, i.e., estimating parameters by minimizing the difference between whole-model output and measured data using an optimization algorithm, has become a common choice.

Intracanopy variability among individual plants is prevalent in crops: even within the same stand of a monoculture under a homogeneous environment, the size, and number of organs (leaves, flowers, internodes, etc.) differ among plants. Challenges arise in how to statistically evaluate the organ-level plant production and how to compare the results with the corresponding model output. Until now, most calibration studies have been dedicated to deterministic plant architectures (Ma et al., 2008) with continuous development or some randomness (Evers and Vos, 2013), which has limited the application of FSPM to a more generic use. With the application of high-throughput phenotyping facilities and intelligent algorithms, architectural data are more accessible than ever, which has enabled the development of a universal analysis method that can be applied to different crops. Among FSPMs, GreenLab is one of the pioneering works in model calibration, and the relevant studies beginning from a simple single stem to stochastic trees, as well as related models, are summarized in Table 1. GreenLab describes plant growth and development according to a set of recurrent equations and adapts to most of the 23 architectural models of Hallé et al. (1978). Most recently, calibration studies for stochastic plants have been performed (Vavitsara et al., 2017; Tondjo et al., 2018), but the analytical basis has not yet been sufficiently presented.

**Table 1:** A summary of the work on FSPM calibration.

			**Deterministic FSPM**	**Stochastic FSPM**	**Other models**
Continuous development	Crops	Single stem	Maize Guo et al., 2006, tomato Dong et al., 2008	
		Branching structure	Wheat Kang et al., 2008a, pepper Ma et al., 2011		Wheat Evers et al., 2007;
		Inflorescence	Arabidopsis Christophe et al., 2008, chrysanthemum Kang et al., 2012	Spilanthes Vavitsara et al., 2017
	Trees			Eucalyptus Diao et al., 2012, development only
Rhythmic development	Seasonal trees	Monocyclic	Poplar Liu et al., 2009	Pine tree Wang et al., 2010	LIGNUM Perttunen et al., 1996
		Polycyclic	Beech tree Letort et al., 2008;	Teak Tondjo et al., 2018	Peach Lopez et al., 2008, apple Costes et al., 2008, L-kiwi Cieslak et al., 2011
	Aseasonal trees			

From a botanical perspective, different plant architectural features can be distinguished from crops to trees such as continuous vs. rhythmic (Barthélémy and Caraglio, 2007). In the continuous case, phytomers are added one by one without a significant rest period, as occurs in many crops and tropical trees. In the rhythmic case, the meristem alternates between extension periods and rest periods, as occurs in many temperate trees. Stochastic axis modeling of continuous and rhythmic developmental features has previously been presented (de Reffye et al., 2012). In this paper, we demonstrate how to calibrate a stochastic FSPM for plants (the case of the GreenLab model) with different developmental features at the whole-model level to estimate the functional and, especially, the sink parameters. The principle is more or less generic as it deals with architectural models that are recognized by other FSPMs. Moreover, a loop between submodels for plant development and growth is common for many FSPMs, although the details of the model differ. The presented approach is applicable for plants or shoots exhibiting regular development in their young stages but not those that have experienced heavy pruning or other artificial interference.

This paper is organized in follows: in section. Materials and methods, the underlying botanical knowledge for the GreenLab model and the components of the mathematical model are presented. section Estimating parameters with organic series presents the computation of the mean organic series and its use to estimate sink parameters. In the section Results, the analytical results for continuous and rhythmic development are verified by Monte-Carlo simulation, and a calibration test is performed both *in silico* and for a real plant. The value and limitations of this work are discussed in section Discussion Conclusions are presented in section Conclusion.

## Materials and methods

### Basis for modeling

#### Botanical knowledge

Apical meristems contribute to axis development by adding new phytomers step by step. This function can be continuous or rhythmic. In the continuous case, phytomers are added one by one without a significant rest period. The cumulative number of phytomers in an axis is generally proportional to the daily sum of temperatures (“thermal time”). Many plants exhibit development following this pattern (tomato, maize, cotton, coffee). In the rhythmic case, the meristem alternates between extension periods and rest periods, with each extension period bearing a *growth unit* (GU) that is a set of phytomers built during that period. As a result, an annual shoot comprises one GU (monocyclic case) or several GUs (polycyclic case). In the monocyclic case, only one GU is produced each year. The extension of GUs usually ends in spring, and the rest period will complete the year. In the polycyclic case, the annual shoot can comprise several GUs. During the rest period of the meristem, a bud is generally built containing embryos of future phytomers.

The GU can be issued from preformation or neoformation. Preformation is common in the case of rhythmic development when a bud is formed during a rest period, as observed in beech or poplar. The flush, or the simultaneous extension of all organs in a bud, generates a GU named the preformed part, which can be followed by several months before the next flush appears. By contrast, the continuous functioning of meristems gives rise to a neoformed part. For some particular tree architectures, such as elm or poplar, this phenomenon occurs immediately after the extension of the preformed part.

Botanists have classified 23 botanical architectural models (Hallé et al., 1978). In the Roux model, the trunk is a monopodial orthotropic axis that shows continuous growth, and the plagiotropic branches are inserted continuously; flowering is lateral on the branches, as in coffee trees. The Rauh model is characterized by rhythmic growth and branching, in which all axes are monopodial and sexuality is lateral, as represented by numerous woody plants such as pine trees. These two architectural models are chosen since they are typical of continuous and rhythmic development.

#### Hypothesis

Regarding plant development, it is hypothesized that the amount of time elapsed between two successive phytomers follows an independent and identical distribution. Thus, the appearance of a new phytomer is regarded as a renewal process, and the number of events during a certain period, or counting variable, is asymptotically normal, which can be approximated by a binomial law for the discrete case (de Reffye et al., 2012; Diao et al., 2012).

Regarding biomass partitioning, it is hypothesized that the biomass of an individual organ is the result of a source-sink balance. Additionally, a common pool hypothesis for biomass allocation (Heuvelink, 1995) is assumed, regardless of the distance from the source to the sink. Based on this, organs of the same age and type share the same amount of resources from the common pool. For trees, this hypothesis actually applies to the modeling of primary growth; the local vigor of branches is expressed as secondary growth, which is linked to the number of leaves (Letort et al., 2008).

### Mathematical model

Being an FSPM, the whole GreenLab model consists of a development model (organogenesis) and a growth model (organ expansion). The stochastic development model for both continuous and rhythmic cases are presented first, starting from the axis (single stem) to a branching structure.

#### Modeling axis development

##### Continuous-development

**Figure 1:**
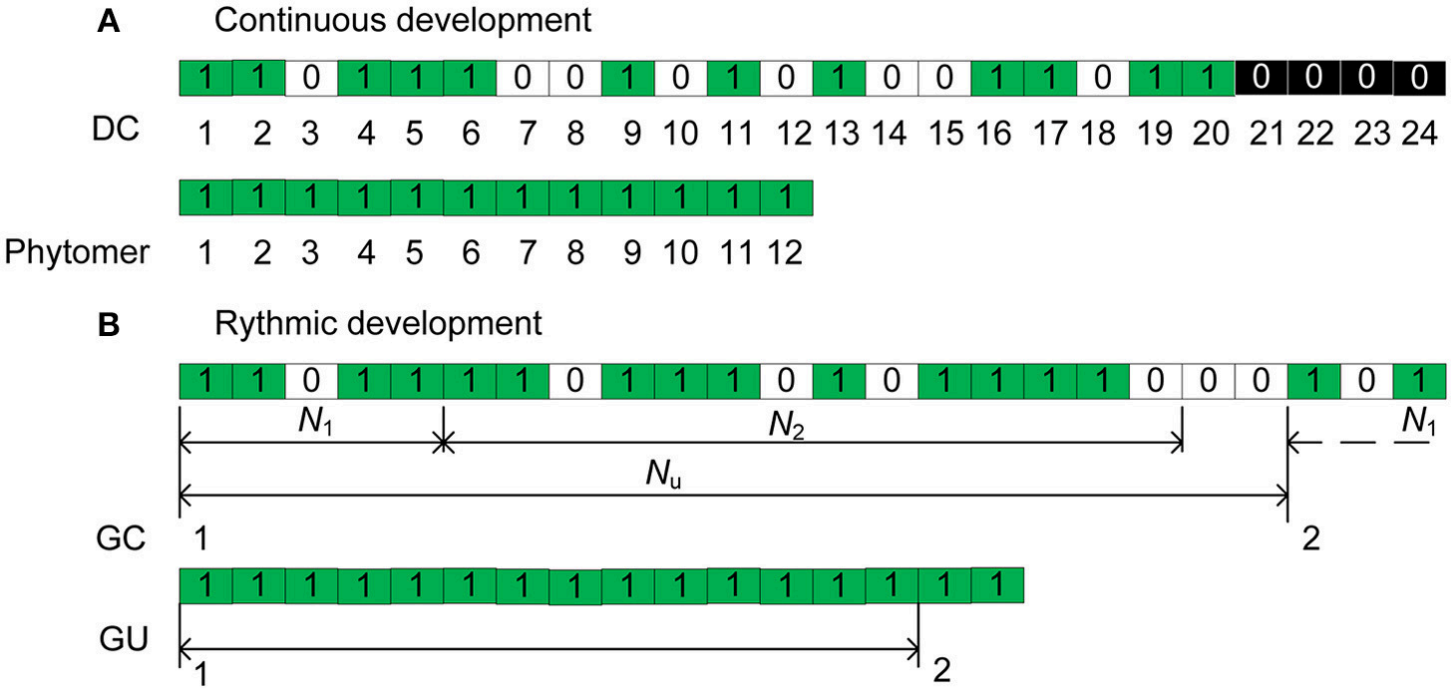
Illustration of the axis development simulation for **(A)** the continuous case and **(B)** the rhythmic case. In **(B)**, a GC is composed of *N*_*u*_ = 21 DCs. For each DC, a green rectangle indicates the creation of a phytomer, an empty rectangle (0) indicates a pause, and a black rectangle indicates the interruption of development. *N*_1_ and *N*_2_ represent the active and pause periods, respectively.

According to de Reffye et al. (2012), the continuous functioning of the terminal meristem of an axis during a certain period can be simulated by a Bernoulli process consisting of discrete development steps. Each step is called a computing unit (CU, de Reffye et al., 2012) or a development cycle (DC) that correspond to the minimal time between the appearances of two successive phytomers. At each DC, a phytomer appears with a development probability *b* (Figure 1A), creating 0/1 series with 0 representing a pause during which no phytomer is born. The chronological age (CA) is defined as the number of DCs that the axis experienced since its appearance. For a given CA of an axis (*n* DCs), the number of phytomers produced follows a binomial law (*n, b*).

Additionally, because of nutrition poverty or insect attack, a reliability probability *c* is introduced to simulate such abortion of meristem activity. Once the mortality of a meristem takes place during a DC, the axis development is interrupted (Figure 1A). The effects of these parameters are explained in more detail in Supplement A. The different aspects of meristem activity are represented by an “axis of development” (de Reffye et al., 2012) that is composed of a series of 1 s and 0s that correspond to the success or failure, respectively, of phytomer production at each DC (Figure 1).

##### Rhythmic-development

Consider the case of an axis with rhythmic development with preformation and neoformation. First, similar to the continuous case, the appearance of a GU can be simulated by a Bernoulli process with a development probability *B*. For the mortality probability c, it is more realistic to consider that death takes place at the end of a GU under rhythmic development.

The total duration of both the extension and rest periods is termed the *growth cycle* (GC). Inside a GC of duration *N*_*u*_ DCs, the meristem produces phytomers during an active period of *N*_1_ DCs and then enters a pause period of *N*_2_ DCs until the end of GC. In Figure 1B for example, the GC duration is *N*_*u*_ = 21 DCs, and that of preformation is *N*_1_ = 5 DCs; 1 GC and 3 DCs are indicated in total. For a GU, according to observations, the numbers of phytomers of the preformed part are distributed according to a positive binomial law (e.g., beech), and the numbers of phytomers of the neoformed part are distributed according to either a positive (e.g., cherry) or negative (e.g., elm) binomial law.

#### Modeling the development of branching structure

Previous approaches for modeling organogenesis include L-system and its extension (Perttunen et al., 1998; Kurth et al., 2005; Costes et al., 2008; Lopez et al., 2008; Cieslak et al., 2011), rewriting rules, reference axis (Barczi et al., 1997) and automaton (Zhao et al., 2003). Considering that we are dealing with both continuous and rhythmic development in this study, the dual-scale automaton is chosen as the organogenesis model (Supplement B). With this botanical automaton, a macro level corresponds to GC/GU, and a micro level corresponds to DC/phytomer. When only one DC is set for a GC, it is a continuous case. Plant development can be programmed by setting different parameters (Zhao et al., 2003; Kang et al., 2008b).

The notion of *physiological age* (PA) is used to classify branch typologies in a hierarchy along a morphological growth gradient. For example, the main stem of a coffee tree has an orthotropic (erect) habit, while the branches are plagiotropic: cuttings from a branch cannot generate a coffee tree, only a creeping plant. The PA of an axis (*p*) is denoted by an integer, with 1 for the main stem, 2 for a first-order branch, etc. The highest PA of the plant is denoted by max*p*. For the Roux architectural model, the PA is almost equivalent to the branching order, except for the reiteration case. It is possible to deduce efficient mathematical operators by counting the number of phytomers of the same type and age, both for deterministic and stochastic cases, without explicitly building the topological structure (Kang et al., 2008b).

Branching probability *a* is used to describe the chance that an axillary bud develops into a branch. With the potential number of buds given for each type of axis, this probability gives different numbers of branches at a branching node. For the rhythmic case (e.g., the Rauh architectural model), there can be multiple phytomers that bear branches of different PA within a GU, and each probability is dependent on the PA of axis *p*. First, the number of DCs that can bear a phytomer (*n*) is drawn according to the chosen binomial law (*N*_1_, *b*), and once *n* is fixed, we draw the branches at random according to the multinomial law (*n, pu*_1, 1_, *pu*_1, 2_, …, *pu*_1, *maxp*_), with *pu*_*p, q*_ being the proportion of branches of PA *q* on an axis of PA *p*. The potential number of axillary branches per GC can be deduced from the parameters *N*_1_, *b* and *pu*_*p, q*_.

#### Modeling plant growth

The functional model of GreenLab is inherited from classical crop models (Brisson et al., 2003) and the accompanying hypotheses of net photosynthesis and light use efficiency. The functional model deals with biomass production (dependent on leaf area), allocation (dependent on sink strength and number of organs) and organ expansion. Accordingly, the computation of biomass production and allocation is tightly linked to the number of individual organs that result from the development model. In the context of stochastic development, Monte-Carlo simulation can provide stochastic samples of plants with the corresponding organ size. This method is very common for stochastic FSPM, and it directly shows the effect of plant development on growth. However, to achieve a good estimation of the population, a large sample is needed, which could be costly. Instead, we expect to obtain the population mean of plants based on the growth of a topologically mean plant. The results are based on equations but not simulations. Such an analytical result is efficient for analyzing the effects of certain parameters on model behavior and, accordingly, for parameter estimation, because in the latter, the procedure for computing the statistical results of the model is performed repetitively. Here, we study both approaches and compare the results. The growth model has been published previously in deterministic form, and here, it is applied without further justification. How stochastic samples for an average plant are computed is shown in Supplement C, and the list of abbreviations and parameters is summarized in Supplement F.

### Derived notions

For convenience in the following demonstration, we introduce several notions related to the stochastic model, which will be used later.

#### Chronological structure

**Figure 2:**
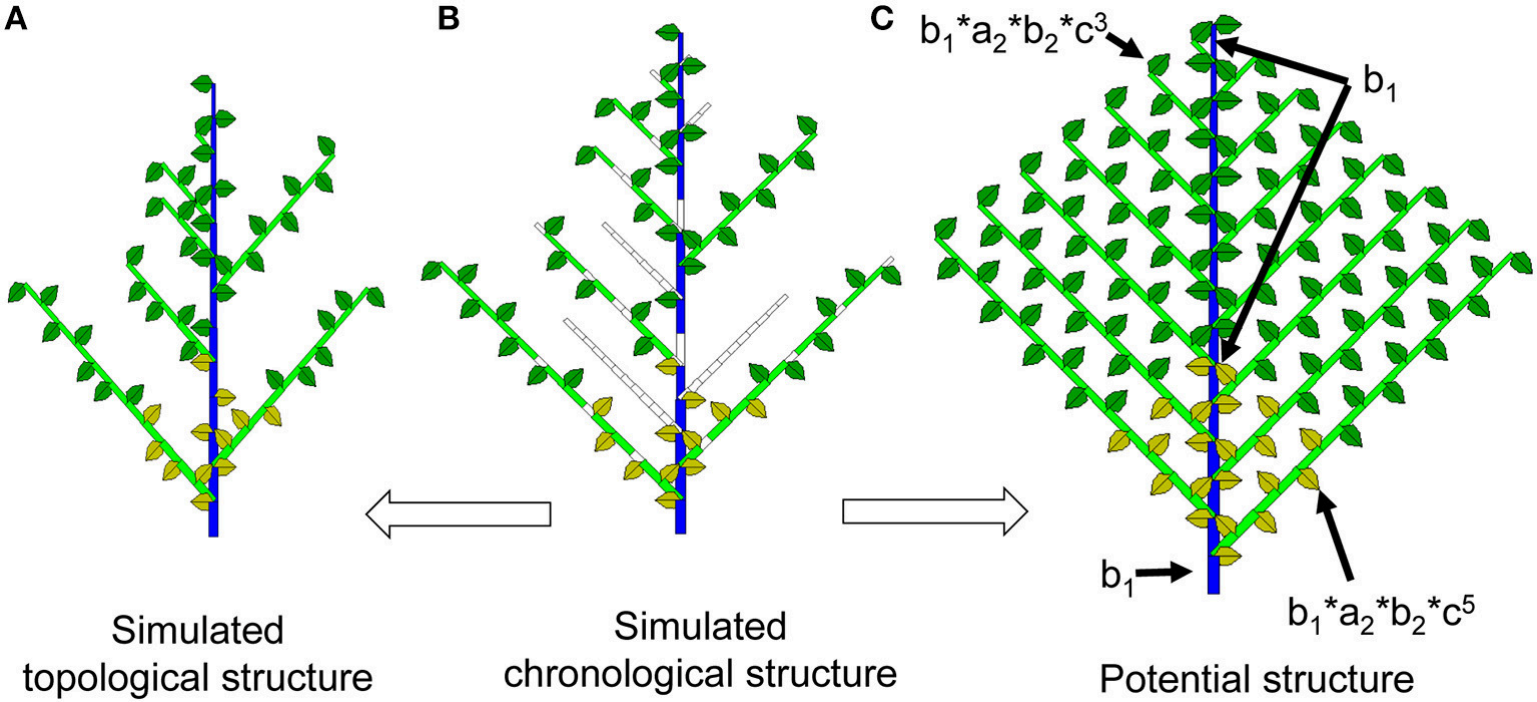
Chronological, topological and potential structures of a Roux architectural model. As a result of Monte-Carlo simulation, the chronological structure **(**middle, **B)** is composed of realized phytomers and void entities. Suppressing the void entities yields the observable topological structure **(**left, **A)**. In the potential structure **(**right, **C)**, each phytomer is associated with a probability of occurrence depending on *a, b* and *c*, which represent the branching, growth, and reliability probabilities, respectively.

Simulating plant development with the Monte-Carlo method produces a series of 1 and 0 values, as shown in Supplement A: 0 represents a temporal pause, and 1 represents the creation of a phytomer. The 0s contain temporal information that is related to the age of the organs. In a Monte-Carlo simulation, it is occasionally interesting to retain these void entities in the displayed plant structure to better understand the effect of bud probabilities. The plant structure retaining the temporal series (both realized and void entities) is called the chronological structure (see Figure 2 for the continuous case). The advantage of this structure is that a phytomer's age can easily be deduced from the rank of the phytomer in the chronological structure. For example, a phytomer of rank 3 from the axis tip has experienced 3 DCs since its appearance.

In the analytical study, the 0s and 1s at each location are replaced by the probability of occurrence of the possible phytomer.

#### Topological structure

The topological structure of plants normally refers to the organization of phytomers in the plant structure (Godin and Caraglio, 1998). In this context, the topological structure refers specifically to the simulated structure containing the realized phytomers, without the temporal entities of 0s as in the chronological structure (Figures 1, 2). The Monte-Carlo simulation of plant uses the actually realized structure. Because of the bud probability, in the topological structure, the age of a phytomer is no longer obvious: for example, a phytomer at rank 3 from the tip can have an age of 7 because of the pause. This finding is similar to the situation in nature, i.e., an organ near the tip can be old and big.

In the analytical study, however, the topological structure is computed from the analytical chronological structure by considering all possible combinations of organ ages.

#### Potential structure and probability of occurrence

The maximum topological structure as defined by the development model is called the potential structure. The potential structure corresponds to the deterministic case when all probabilities are equal to 1 and can be understood as the upper bound of the structure. However, the size of organs in the potential structure is dependent on the bud probabilities and the source and sink parameters.

Each potential phytomer site is characterized by a *probability of occurrence*, which is the probability that a phytomer appears at this temporal site. In the Monte-Carlo simulation, this probability can be estimated as the average of the 1s and 0s at the same site of the chronological structures through numerous simulations.

In the analytical study, each phytomer site is attributed an analytical probability according to the bud probabilities. The sum of all phytomer probabilities in the potential structure defines the mean number of phytomers produced by the structure. The numbers of each organ type (leaf, internode, and fruit) by PA are known on a given phytomer, thus providing the mean organ production, from which the mean plant demand can be defined. The growth of this conceptual structure produces an analytical plant that is difficult to realize by simulation.

### Organic series

An *organic series* was originally defined as the dimension or weight of organs produced sequentially along an axis of development (Buis and Barthou, 1984), and this architectural characteristic contains rich information about the growth of a plant. Such information has been used to describe the plant profile and for parameter estimation in deterministic FSPM (Ma et al., 2008). In the stochastic model, there are variations in organic series of the same type with the same parameters; thus, an average organic series is expected. For the axis of the same PA, since organs of the same PA and CA have the same biomass partitioning according to the model hypothesis, one organic series is sufficient for a given PA. Considering the number of organ types (*t*) in the axis, in total, there are max*p* × *t* organic series for a plant.

In the Monte-Carlo simulation, for a chronological organic series, each component is the average value of organ size including empty phytomers. The topological organic series is defined as the average organ size of phytomers of the same rank from the top or bottom of the observed axis. In the analytical study, an organic series is computed by growing the potential plant structure with the probability of occurrence for each phytomer.

### Software implementation

The simulation and computation experiments were performed in the software “Gloups” developed under the Windows environment using MATLAB (The MathWorks, Inc., America). The main procedures include one for simulation and another for calibration. In the simulation, the Monte-Carlo method is applied for stochastic development. The results are shown for different plant samples. A virtual target data file can be output. In the calibration, the analytical computation of the organic series is performed according to the initial parameter values, and the result is fit with the target data, either from simulation or from real plants. A simple tutorial is shown in Supplement E.

## Estimating parameters with organic series

### Organic series for shoots with continuous development

#### Probability of occurrence

For plants with continuous development, the probability of occurrence is a compound result of the probabilities of branching, growth, and reliability (*a, b*, and *c*), respectively (Figure 2). For example, for the potential phytomer of PA 1 that appeared at the *i*^th^ DC, its occurrence probability is *b*_1_*c*${}_{1}^{i}$.

#### Analytical demand

The potential structure with its corresponding probability of occurrence represents an analytical average plant that does not exist. Computing the growth of potential structure gives a measure of an average plant. In the Monte-Carlo simulation, a superscript *s* is used to indicate the variable of random plant sample; see Supplementary C. By contrast, in the analytical study, the variables are denoted by a superscript θ, such as *Q*^θ^(*n*). The computation of *Q*^θ^(*n*) shares the same formula as that for stochastic samples. For the analytical demand, the equation's form is different from that for stochastic simulation and is computed by replacing the number of organs with the corresponding probabilities of occurrence in a chronological series:

(1) $$\begin{array}{r} D^{\theta }\left(n\right)=\overset{M}{\underset{id=1}{\sum }}\underset{o}{\sum }\left(\pi \left(id\right)\cdot P_{o}^{p}\left(i\right)\right) \end{array}$$

where *M* is the total number of phytomers in the potential structure; *n* is the plant age; π(*id*) is the compound probability of occurrence of phytomer *id*, depending on its position in the plant structure; *P*_*o*_ is the sink strength of the organ; *p* is its physiological age; and *i* is the number of DCs experienced since its expansion.

Accordingly, the biomass increment for the organ *o* at age *i* is:

(2) $$\begin{array}{r} \Delta q_{o}^{p,\theta }\left(i,\;n\right)=P_{o}^{p}\left(i\right)\frac{Q^{\theta }\left(n-1\right)}{D^{\theta }\left(n\right)} \end{array}$$

#### Analytical chronological series

Accumulating the biomass increment in each cycle gives the analytical chronological organic series $\left[q_{o}^{p,\theta }(*,\;n)\right]$ of organ *o*, as in (3).

(3) $$\begin{array}{r} \left[\begin{array}{c} q_{o}^{p,\theta }\left(1,n\right)\\ q_{o}^{p,\theta }\left(2,n\right)\\ \vdots \\ q_{o}^{p,\theta }\left(n,n\right) \end{array}\right]=\left[\begin{array}{ccc} P_{p}^{o}\left(1\right) & 0 & \begin{array}{cc} \cdots & 0 \end{array}\\ P_{p}^{o}\left(2\right) & P_{p}^{o}\left(1\right) & \begin{array}{cc} \cdots & 0 \end{array}\\ \begin{array}{c} \vdots \\ P_{p}^{o}\left(n\right) \end{array} & \begin{array}{c} \vdots \\ P_{p}^{o}\left(n-1\right) \end{array} & \begin{array}{c} \ddots \\ \cdots \end{array}\;\begin{array}{c} \vdots \\ P_{p}^{o}\left(1\right) \end{array} \end{array}\right]\left[\begin{array}{c} \frac{Q_{n-1}^{\theta }}{D_{n}^{\theta }}\\ \vdots \\ \begin{array}{c} \frac{Q_{1}^{\theta }}{D_{2}^{\theta }}\\ \frac{Q_{0}^{\theta }}{D_{1}^{\theta }} \end{array} \end{array}\right] \end{array}$$

If the reliability probability *c* is high, the number of organs in plants are mainly the result of a *Bernoulli* process, and the simulated *Q*^*s*^*(t)* and *D*^*s*^*(t)* are roughly symmetrically distributed. In this case, the following approximation can be properly obtained as verified by stochastic simulations:

(4) $$\begin{array}{r} \overset{\bar{}}{{q_{o}^{s}\left(i,n\right)}_{ch}}\approx q_{o}^{\theta }{\left(i,n\right)}_{ch} \end{array}$$

where *i* is the CA of organs; *o* represents the organ type; and ch refers to the data of chronological organic series. As mentioned, superscripts *s* and θ represent simulated (Supplementary Equation S5) and analytical results, respectively.

#### Analytical topological series

The analytical topological series (indexed with tp) is computed to provide a model result equivalent to the measured data. The series is computed from the chronological series by converting the age to position. Considering a phytomer of rank *K* from the tip of a living axis, the possible CA (*i*) of this phytomer is *K, K*+1, …, *n* because of the *Bernoulli* process. Accordingly, the probability that a phytomer of rank *K* from the tip has a CA *i*, $\underset{K}{\mathrm{Pr}}\left(i\right)$, follows a truncated negative binomial distribution (*K, b*) as follows:

(5) $$Pr_{K}(i)=\{\begin{array}{cc} C_{n}^{i}\cdot b^{i-K}\cdot {\left(1-b\right)}^{K} & K\leq i<n\\ 1-\overset{j=K}{\underset{n}{\sum^{\text{​}}}}C_{n}^{j-1}\cdot b^{j-K}\cdot {\left(1-b\right)}^{K} & i=n \end{array}$$

Given the organ age, the weight of the organ is defined in chronological series. Then, the average organ weight located at rank *K* from the axis tip, ${q_{o}^{p,\theta }\left(K,n\right)}_{tp}$, is a compound sum of organs with age *i* (*K* ≤ *i* ≤ *n*), ${q_{o}^{\theta }\left(i,n\right)}_{ch}$, computed in a chronological structure.

(6) $$\begin{array}{r} {q_{o}^{p,\theta }\left(K,n\right)}_{tp}=\frac{\overset{n}{\underset{i=K}{\sum }}{Pr}_{K}\left(i\right)\cdot q_{o}^{p,\theta }{\left(i,n\right)}_{ch}}{\overset{n}{\underset{i=K}{\sum }}{Pr}_{K}\left(i\right)} \end{array}$$

In the Monte-Carlo simulation, the average organ weight at a given phytomer rank from the axis tip, $\overset{\bar{}}{{q_{o}^{p}\left(K,n\right)}_{tp}}$, is computed directly from the simulated samples $q_{o}^{p,s}{\left(K,n\right)}_{tp}$:

(7) $$\begin{array}{r} \bar{{q_{o}^{p}\left(K,n\right)}_{tp}}=\frac{1}{T}\overset{T}{\underset{s=1}{\sum }}q_{o}^{p,s}{\left(K,n\right)}_{tp} \end{array}$$

where *T* is the number of simulated samples. Although one is from stochastic simulations and the other is from analytical computations, the following equivalence holds:

(8) $$\begin{array}{r} {\bar{q_{o}\left(K,n\right)}}_{tp}\approx q_{o}^{p,\;\theta }{\left(K,n\right)}_{tp} \end{array}$$

In summary, the analytical mean organic series can be obtained by ‘growing' an average plant. The results can be verified by the results of the Monte-Carlo simulation to provide an organ-level measure of a stochastic FSPM for comparison with experimental data, which is the basis for parameter estimation.

### Organic series for shoots with rhythmic development

#### Probability of occurrence

Rhythmic development is an extension of the continuous case in which each GU is regarded as a phytomer. The existence of a phytomer is jointly decided by GC (macro level) and DC (micro level).

##### GC-level

**Figure 3:**
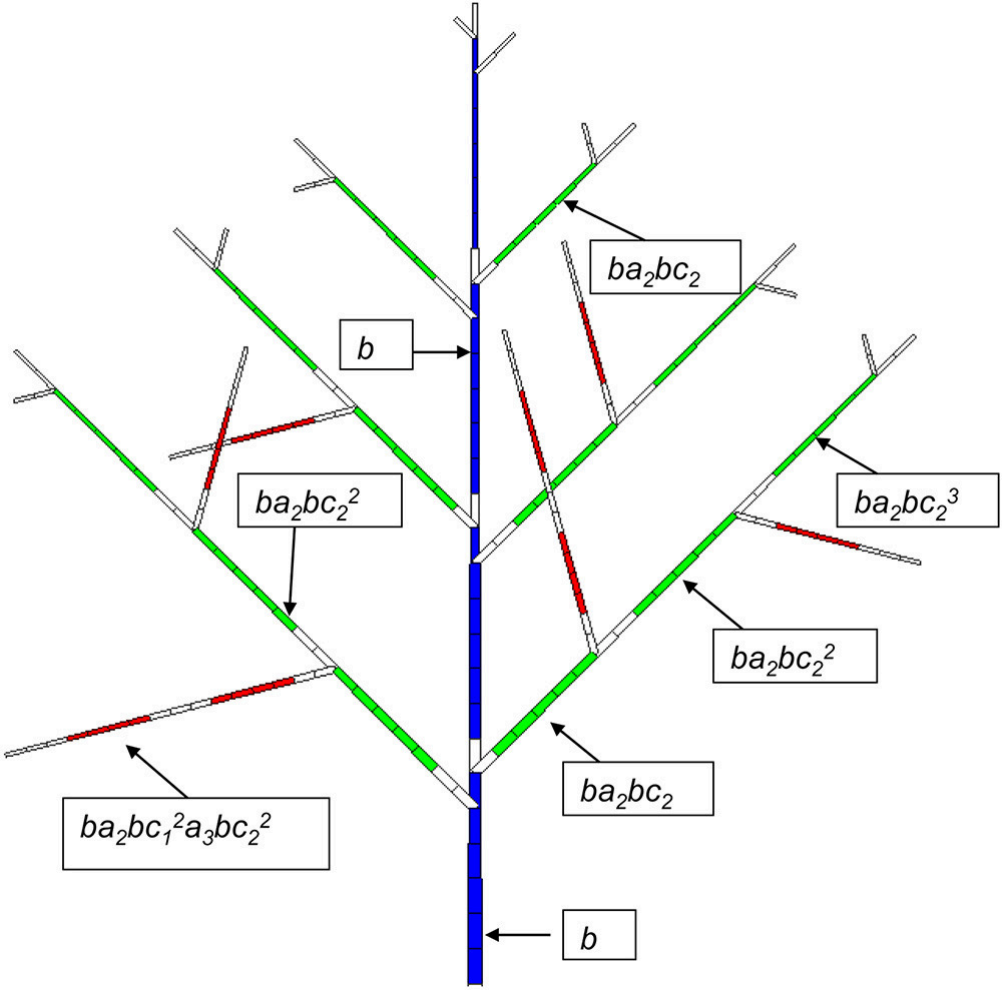
Potential structure of a Rauh architectural model with a synchronous structure. A GC is composed of 7 DCs. The age of the plant is 4 GCs (28 DCs). The development period is 6 DCs for PA 1 (blue), 5 DCs for PA 2 (green) and 4 DCs for PA 3. The axes of PA 2 and PA 3 have a probability of branching of *a*_2_ and *a*_3_, respectively, and the viability of the meristems at the GU level in the axes is *c*_2_ and *c*_3_, respectively. *b* is the occurrence probability of a phytomer. An empty rectangle indicates a pause.

The probability of occurrence of a GU is similar to that of a phytomer in the continuous case. It can depend on the *Bernoulli* process of bearing a GU (probability *B*), the sequence of ramifications (probabilities *a*), and the succession of GUs along the axes with probability *c*. Figure 3 shows a relatively simple case where *B* = 1, which means at each year a GU is produced.

##### DC-level

If a GU exists inside this GC, phytomers at each DC are simulated with a *Bernoulli* process, as in the continuous case. In generic case both pre- and neoformation exist inside a GU. Since the number of phytomers in the preformation part usually follows a binomial distribution, say (*N*_1_, *b*), a fixed duration of development (*N*_1_) is set for this period, as is the case of Figure 3. The distribution of the number of DCs in this preformation period, x_1_, can thus be expressed as follows:

(9) $$P(x_{1}=i)=\{\begin{array}{c} 0\;(i\neq N_{1})\\ 1\;(i=N_{1}) \end{array}$$

The number of DCs in the neoformation period (*x*_2_) follows another distribution (*F*) that is either a positive or negative binomial distribution. Moreover, since the neoformation part does not always exist, there is a passage probability (*d*) from preformation to neoformation correspondig to period *N*_*d*_. Finally, the distribution of having a duration of *i* DCs for axis development, *G*(*N*_*d*_), is a mixture of *P* and *F* distributions.

(10) $$\begin{array}{r} G\left(N_{d}=i\right)=\left(1-d\right)\cdot P\left(x_{1}=i\right)+d\cdot \overset{i}{\underset{k=1}{\sum }}P\left(x_{1}=k\right)\cdot F\left(x_{2}=i-k\right) \end{array}$$

This distribution can infer the probability that this GU continues organogenesis at the *i*^th^ DC from its beginning, by summing all the probabilities of having less than *i* GCs for development (*N*_*d*_<*i*). Like the continuous case, if there is a probability (*b*) of making a phytomer at each DC, independent of the distribution of the number of DCs in pre- and neoformation, one obtains a law that is between the development times and the *Bernoulli* process. The probability of the occurrence of the phytomer at the *i*^th^ DC in a GU is finally written as:

(11) $$\begin{array}{r} b_{i}=b\cdot \left(1-\overset{i-1}{\underset{j=1}{\sum }}G\left(N_{d}=j\right)\right) \end{array}$$

The product of the occurrence probability of a GU (similar to the continuous case) and that of a phytomer in the GU gives the final occurrence probability of each potential phytomer.

#### Analytical demand

Therefore, the development axis is constructed from the periodic sequence of GCs, in which the phytomers appear with probabilities *b*_*i*_. The potential structure is constructed by assembling the axes according to branching rules. As mentioned, for branching of the axis of PA *p*, we choose a multinomial law *(N*_*d*_, *pu*_*p, p*_, *pu*_*p, p*+1_,…, *pu*_*p, maxp*_*)* so that the proportions among different types of branches remain constant when *N*_*d*_ varies according to the compound law. In simulation, the branches are drawn and sorted in acrobatic or basitonic order. The potential number of axillary branches per GU is deduced from these parameters. For example, the potential number of branches of PA 2 on GU of PA 1 is *N*_1_^*^pu_*p, q*_.

The probability of occurrence at each DC is the product of the probabilities of GU and phytomer, i.e., P${}_{\text{GU}}^{*}$b_*i*_. Accordingly, for a GU born at GC *x*, its analytical demand from all potential phytomers, ${D_{GU}}^{\theta }\left(x\right)$, is written as the following:

(12) $$\begin{array}{r} {D_{GU}}^{\theta }\left(x\right)=\underset{o}{\sum }\overset{Nu}{\underset{i=1}{\sum }}P_{GU}\left(x\right)\cdot b_{i}\cdot P_{o}\left(N_{u}-i\right) \end{array}$$

where *P*_*GU*_(*x*) is the occurrence probability of a GU and *P*_*o*_(*N*_*u*_−*i*) is the sink strength of the organ that appears at the *i*^th^ DC in a GU. It is therefore possible to calculate the total demand of a plant by summing the demand of all potential GUs. Similarly, by “growing” the potential plant, one can obtain the analytical chronological series, whose data are organized at both the macro and micro level.

### Estimating parameters for organ growth

**Figure 4:**
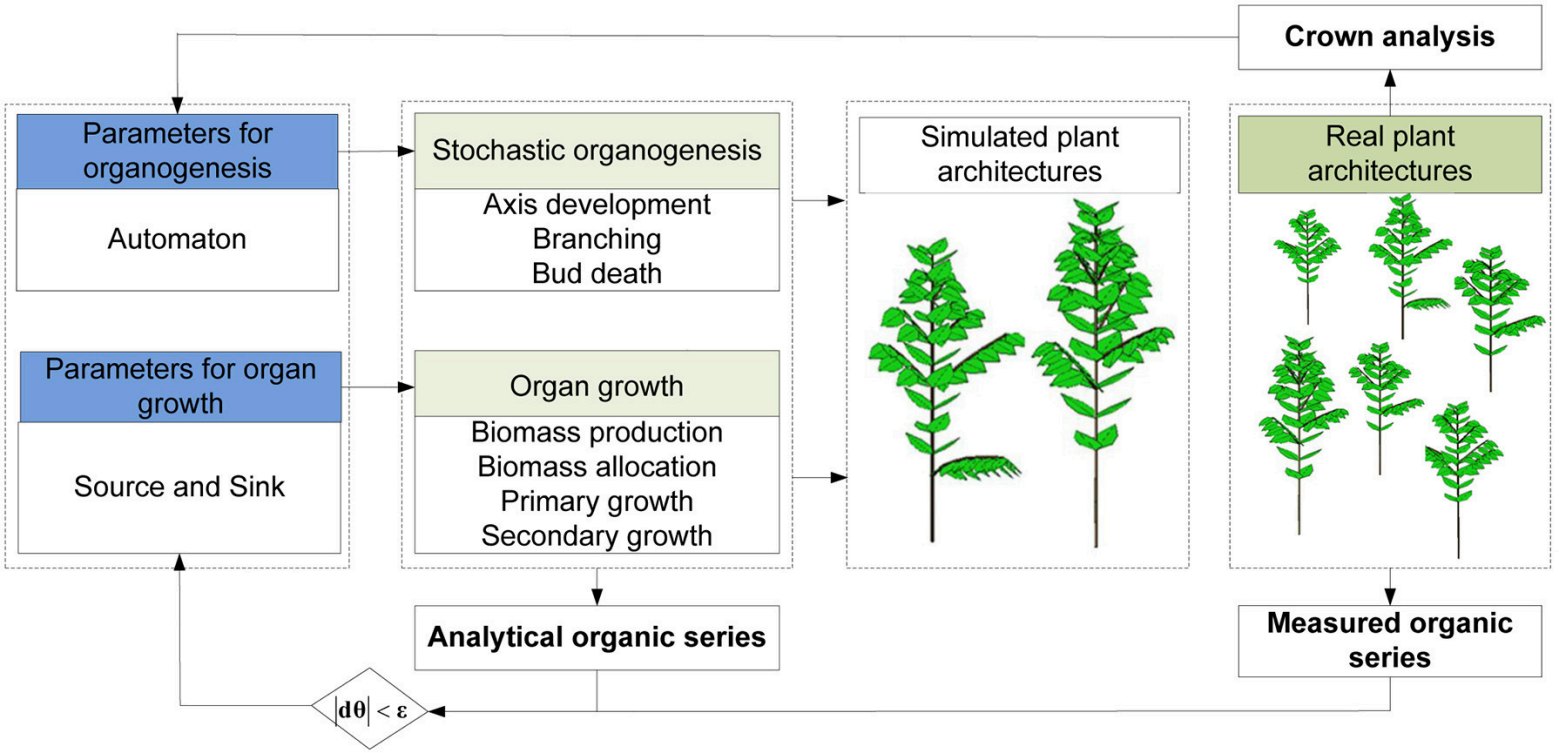
The framework used for estimating parameters for a stochastic functional-structural plant model.

Since an FSPM basically consists of two parts: a module for organ production (organogenesis) and a module for organ growth, the key model parameters can correspondingly be divided into two parts. Figure 4 lists the general framework for parameter estimation. First, plant crown analysis (Diao et al., 2012) (top-down statistical analysis of the number of phytomers in plant axis pairs) is used to estimate parameters for organogenesis, including the development probabilities (*b* and *B*), reliability probability (*c*) and branching probability (*a*). The macrolevel analysis for the rhythmic case is similar to that for the continuous case. Inside a GU, the distributions for pre- or neoformation can be estimated by counting the number of phytomers. Second, parameters for organ growth, which control the functional model including the source and sink parameters, are identified by fitting the measured organic series with their analytical correspondents.

As previously mentioned, the estimation of organ functioning parameters from the plant architecture measurements has been worked out for plants with deterministic development (single-stem plants), such as maize (Guo et al., 2006) and beetroot (Lemaire et al., 2009). The target for fitting includes max*p* types of axes (max*p* being the largest PA) and *t* types of organs. In total, there are max*p* × *t* organic series, which are few. For example, for a plant population of the Roux model with only two types of organs (leaves and internodes), although each plant may potentially bear approximately 20 first-order branches, in total four organic series are sufficient for describing the population: two for the main stem organs and two for the branch organs.

Source and sink parameters are inversely estimated by fitting the observed statistical organic series with the analytical model output. An optimization algorithm is applied to find the best set of parameter values that can minimize the difference between the analytical and measured topological organic series for all organ types and measurement dates.

(13) $$|dif|=\underset{o,p,n}{\sum^{\text{​}}}|{\left[q_{o}^{p}(*,\;n)\right]}_{tp}-{\left[q_{o}^{p,\theta }(*,n)\right]}_{tp}{|}_{n}$$

where ${\left[q_{o}^{p,\theta }(*,\;n)\right]}_{tp}$ is computed in (6). For real plants, ${\left[q_{o}^{p,\theta }(*,\;n)\right]}_{tp}$ is the average weight of organs ranked from the top of the plant crown, without recording the full plant topological structure as previously done (Kang et al., 2008a; Letort et al., 2008), which is very practical. We use the nonlinear generalized least squares method to achieve the estimations. The principle is similar to the deterministic case, but the measure accounts for the intervariability.

## Results

FSPMs are generally very complex, and software implementing FSPMs must be carefully tested to give sound numerical results. It is relatively easy to obtain a plausibly reasonable plant structure, but for parameter estimation and model analysis, proper results are of the highest importance. Monte-Carlo simulations of plants provide two types of results concerning both development and growth:

- Stochastic plant crowns, which are outputs of plant development and implicitly contain the parameter values of the meristem functioning;
- Stochastic organic series, which are outputs of plant growth and implicitly contain the values of the sink-source parameters of the organ functioning.

The simulation result itself is noisy, and the statistical precision is dependent on sample size. However, its value lies in (1) providing a way to verify the analytical result, which is computed with an independent procedure, and (2) providing virtual plant samples for *in silico* fitting before use with real plants of the same type. For the second purpose, the advantage is that the actual developmental and functional parameters are known; thus, whether the fitting process works properly can be tested. The results are organized as follows: first, the test of parameter estimation is shown for plants with the continuous case for both virtual and real plants. The case covers all bud probabilities mentioned above. Then, the test result is shown for the case of rhythmic development.

### Case of continuous development

Set the development parameters for the Roux model with two PAs as *b*_1_ = 0.8, *b*_2_ = 0.8, *a*_2_ = 0.9 and *c*_2_ = 0.95 (constant). The main stem has no abortion (its reliability probability *c*_1_ is set to 1). Let *CA* of the plant be 30 DCs. Let the sink strength be identical for leaves and internodes. The expansion function of organs *F*_*o*_ is set to an even distribution.

#### Test *in silico*

##### Showing-the-derived-concepts-on-a-single-stem-plant

**Figure 5:**
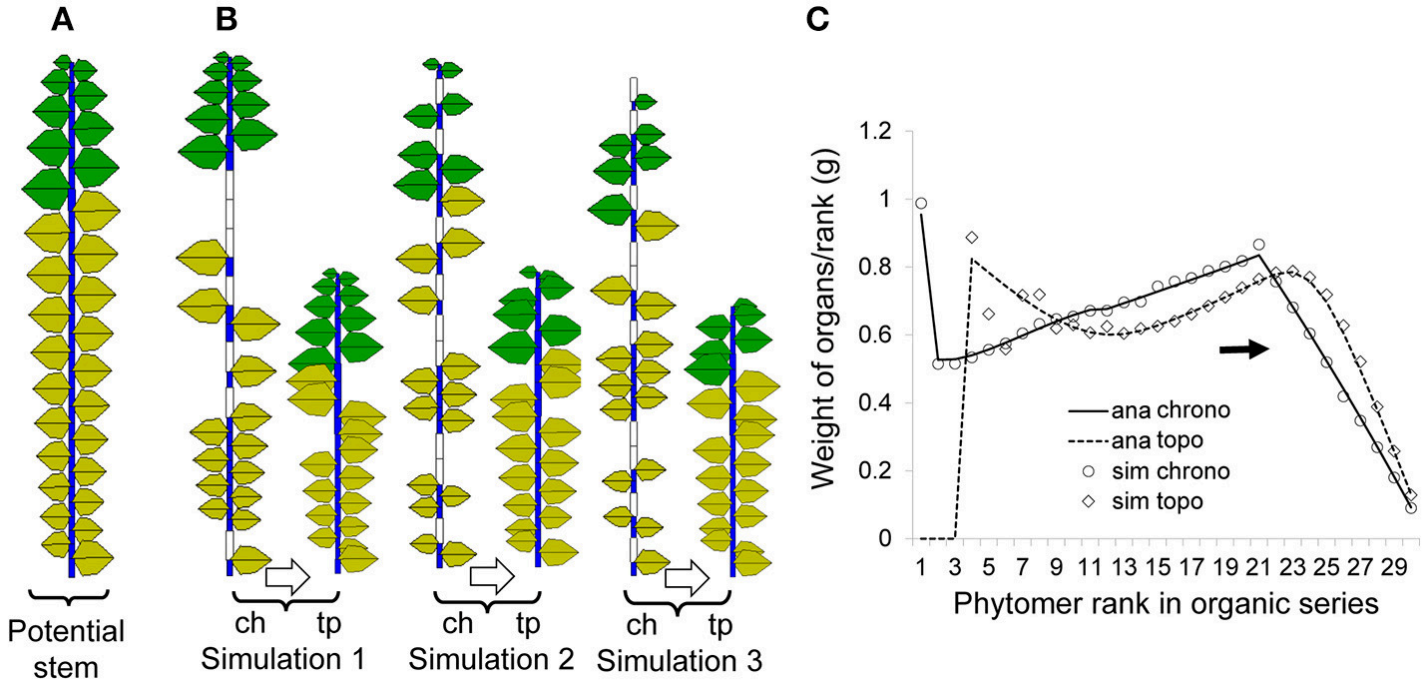
Potential, simulated and analytical (computed) chronological and topological organic series for a single stem developed from a *Bernoulli* process (*b* = 0.7, *n* = 30) with 1000 simulations. The left **(A)** is the potential structure followed by three pairs of stochastic simulations **(B)** in chronological mode (*ch*) and the corresponding topological mode (*tp*); yellow leaves are no longer functional. The right **(C)** shows the corresponding curves for the organic series sorted from the stem tip (right side); the analytical results (lines) and results from simulations (symbols) are both given for comparison.

To provide a clear understanding of the mentioned concept, focus is first given to a single-stem plant (by setting max*p* = 1). Both the functioning and expansion durations of organs are set to 5 DCs. Figure 5A shows a potential structure containing 30 phytomers, the same number as the plant CA. Figure 5B shows three pairs of chronological and topological structures from the Monte-Carlo simulation: at left (“ch,” chronological structure), blank entities are inserted to show a pause, and in total, they always contain 30 entities. At right (“tp,” topological structure), the actual simulated entities are shown.

Figure 5C contains rich information, which is helpful to understand the difference between the chronological and topological series in a numerical way. As the chosen expansion function is linear, the top part in the chronological series (solid line) is linear as well. However, when the results are converted to the topological series (dotted line), the top part is no longer linear since each data point is a combination of those from the chronological series.

The average organic series from the simulation is shown in symbols and matches well with the analytical data. Note that this is not fitting but a comparison of the results from the analytical computation using the potential structure with the results from the Monte-Carlo simulation.

##### Parameter-estimation-test-in-the-roux-architectural-model

In this example, the leaf functioning time, *t*_*a*_, is set to 9 cycles. The expansion time of organ *t*_*x*_ is set to 9 cycles as well. The specific leaf weight, *e*, is set to 0.05 g cm^−2^. Sinks for all organs are set to be equal for the internode and leaf and for the main stem and branch, with *p*_*o*_ = 1; the light use efficiency (*r*) for leaves is set to 30; and the production surface, *S*_P_, is set to 2000 cm^2^.

**Figure 6:**
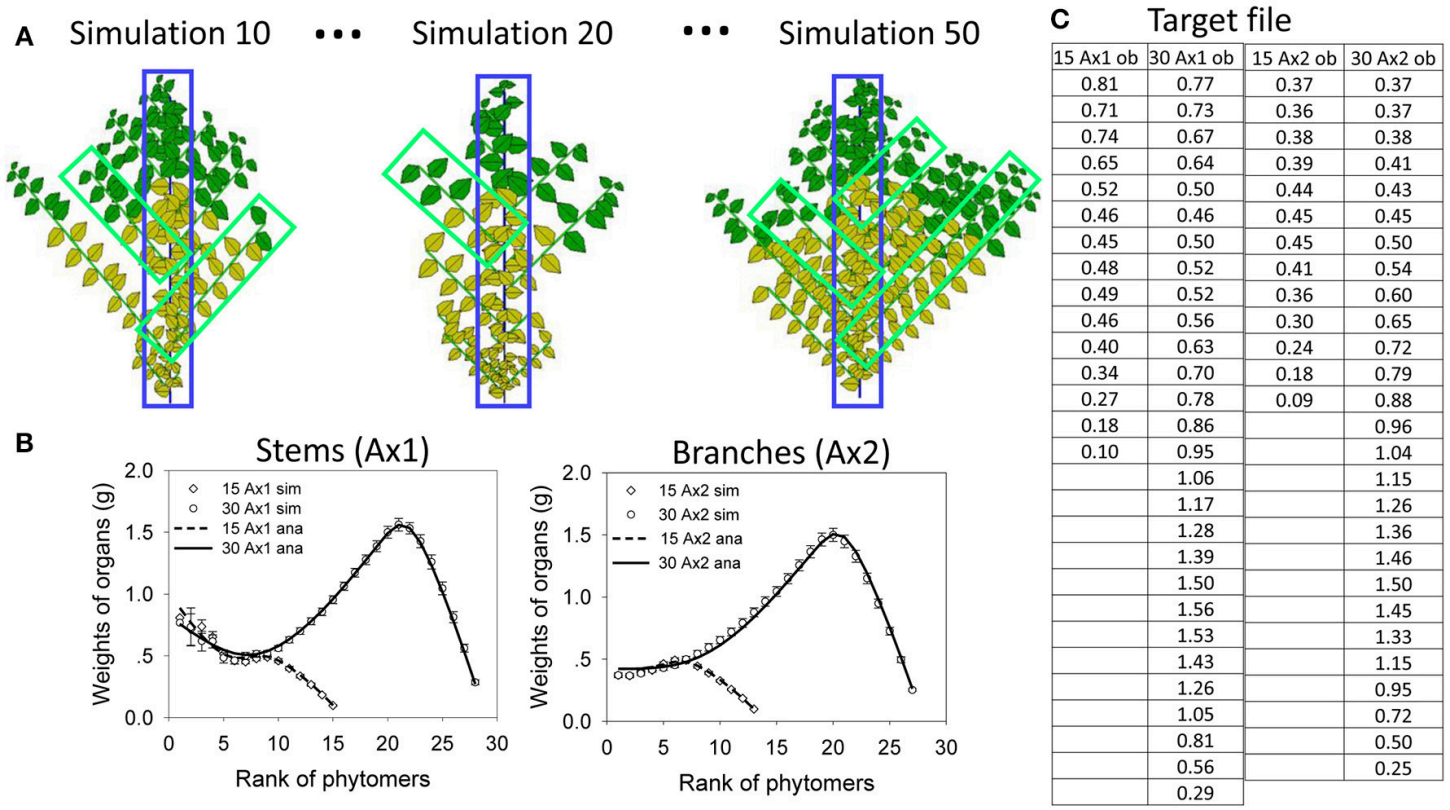
*In silico* parameter estimation for plants with continuous development. Top left **(A)**: simulated plant samples for selecting organic series for stems (blue) and branches (green). Bottom left **(B)** the fitting curves between the average organic series taken from the simulation (circles and squares, bars showing standard deviation) and the analytical series (solid and dotted lines) at DC 15 and 30, for main stem (Ax1) and branches (Ax2). The table **(C, right)** shows the target data for topological organic series at two ages (15 and 30 cycles) on the main stem (Ax1) and branches (Ax2).

The target data for fitting (Figure 6A) are the result of virtual sampling of the simulated plants (illustrated in Figure 6B). Figure 6A shows data at age 15 and 30. At each age, there are a total of four (topological) organic series: two for the stem (leaf and internode) and two for the branches (leaves and internodes). Since the leaf and internode share the same sink functions, their organ series are simplified as the same. Therefore, at each plant age, only two series are considered: one for the stem and one for the branches. Figure 6B shows three plant samples from 50 simulations. The samples for branch can be “picked” from different plants according to their rank. The dead branches with all yellow leaves are omitted. The branches are then averaged from the top to bottom to obtain average organic series for the branches. This simulates the action in a real experiment. It is not necessary to sample all branches.

Figure 6C show the results of the fitting test, using the data from virtual samples to fit the target. The standard deviations of the average data are shown as well. The virtual sample data are fit by the analytical results computed directly from parameter values. The hidden parameters to be The standard deviations of the average the target data are *r, S*_P_, *p*_*a*_ for the leaf and *p*_*i*_ for the internode. Starting with initial values with a 10% deviation from the real values, the nonlinear generalized least squares method yields stable solutions with *r* = 29.7, *S*_P_ = 1978 cm^2^, *p*_*a*_ = 0.96**,** and *p*_*i*_ = 0.98. These values are quite similar to the original data used to simulate the stochastic plant. The fitting of the average organic series with the analytical results is processed simultaneously for the two growth stages, which is called multifitting. The fitting curves are shown in Figure 6, which reveals that the analytical organic series fits well the corresponding average organic series. The time of parameter estimation is only tens of seconds. Moreover, the time depends on the initial parameter values for real plants.

#### Test on real plants: case of young coffee trees

A recent publication on the application in real plants is Vavitsara et al. (2017). Here, we present another study of the coffee tree that was conducted in Ivory Coast by Sélastique Akaffou (University Jean Lorougnon Guédé, Ivory Coast) with a collection of young coffee trees. The analysis was done through 14 repetitions of the same plant (clones) of the same age. These young plants did not yet show meristem abortion or fruitage.

##### Plant-material

The coffee tree is a woody plant with stochastic development and continuous growth. Phytomers bear two leaves and potentially two axillary meristems (on the stem). Plant architecture follows the Roux model with orthotropic stems and plagiotropic branches that yields two PAs and two kinds of organic series (leaves, internodes) for each PA. Note that woody plants have girth growth. How the GreenLab model identifies the parameters of girth growth is demonstrated in Kang et al. (2002).

Fourteen young coffee trees of the same age belonging to the species *Coffea pseudozanguebariae* were cultivated in a stand under homogeneous conditions. This widespread species is studied since it is naturally caffeine-free and provides the opportunity to produce a low-caffeine or caffeine-free variety of the cultivated species *Coffea canephora* (Robusta). The organs used to build organic series of stems and branches were measured in dry weights.

##### Estimating-development-parameters

**Figure 7:**
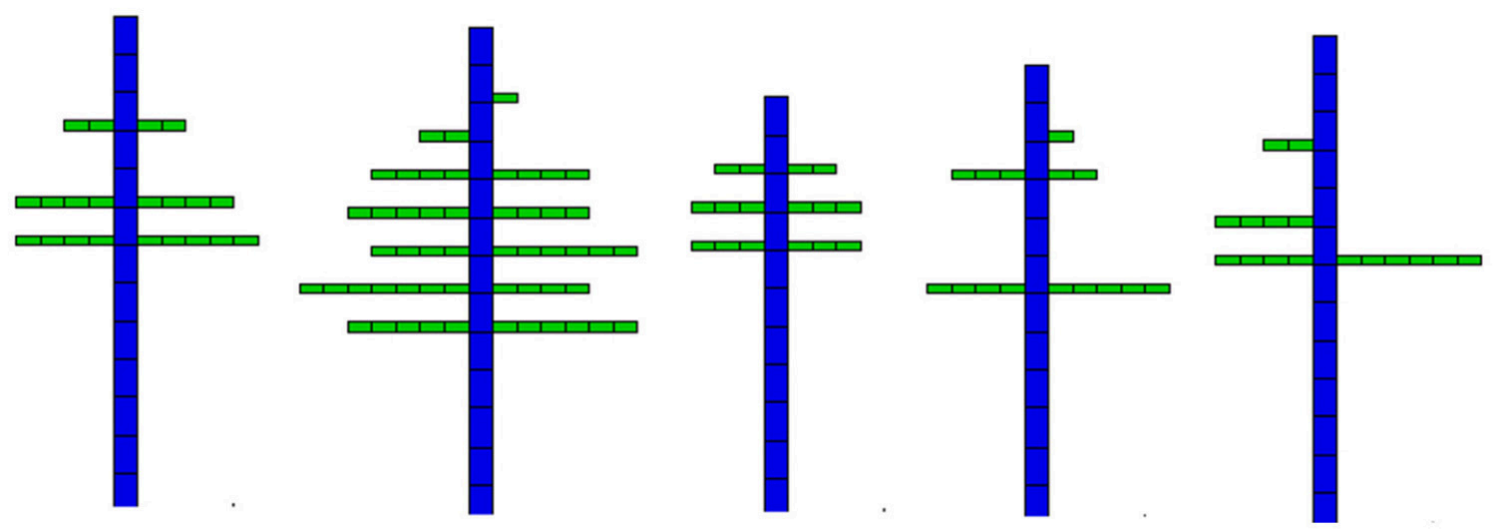
Five topological structures of coffee trees.

Figure 7 shows five observed topological structures among the 14 coffee trees included in the data sets. One can see the results of the stochastic development. Branches are always missing at the bottom, and the ramification rate increases with plant development. The Bernoulli process is visible. Two adjacent branches belonging to the same whorl on the stem can have different phytomer numbers.

**Figure 8:**
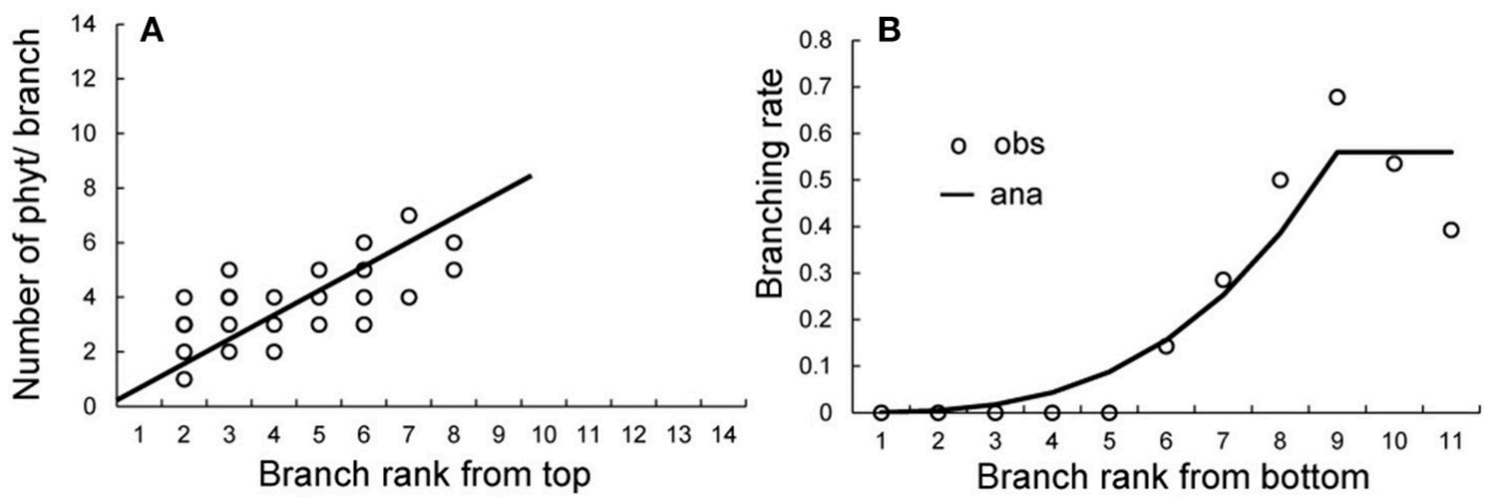
Obtaining development probabilities in tree crown analysis. **(A)** Fitting of the number of phytomers in branches at rank *K* from the stem top to give the development probability *b*. **(B)** Fitting of the branching rate at each rank from the bottom of the stem to give the variable branching probability *a*. Circles: observed data; solid lines: fitting results.

The crown analysis retrieves the development parameters (*a, b*, and *c*) from the plant architecture measurements (Diao et al., 2012). Fitting is performed independently to retrieve different probabilities. The crown analyses for obtaining bud probabilities are shown in Supplement D. Figure 8A shows the effect of the *Bernoulli* process on the size of the branches at rank *K* from the stem tip. Once the target file provided by the tree crowns is built, we can solve the equation (S15) in Supplement D to assess the parameters *b*_1_, *b*_2_ and *w*, and we obtain *b*_1_ = 0.8, *b*_2_ = 0.9, and *w* = 0.75.

The coffee tree exhibits immediate branching, and missing branches are the result of early axillary meristem abortion. The rates of ramification increase from the bottom of the stem to achieve stabilization after the rank *K* following the equation:

(14) $$\begin{array}{r} a_{K}=0.62\cdot {\left(\frac{K}{9}\right)}^{3.45},\;K\leq 9 \end{array}$$

$$\begin{array}{r} a_{K}=0.62,\;K\leq 9 \end{array}$$

Beyond the rank 9 from the bottom, the ramification rate stabilizes to *a* = 0.62 (Figure 8B).

These values that control the meristem functioning allow stochastic simulations of plant development as well as the potential structure to be built.

##### Organic-series-analysis

There are 4 types of organic series (leaves and internodes) for both stems and branches. Averages of organ dry weights according to the rank from the top of living axes generate the organic series, and these observed series are fitted to the analytical ones computed from the potential structure as previously worked out. The target file for coffee trees is constructed for a CA of 16 DCs. Some parameters can be assessed from direct measurements, and the others are hidden and estimated by the inverse method.

*Parameters from direct measurements* The parameters are functioning times and allometries. Leaf functioning time is 12 DCs, and the expansion times of leaves and internodes are 4 and 3 DCs, respectively. The specific leaf weight, *e*, is 0.016 g cm^−2^, and the sink strength of the stem leaves is conventionally set to 1. The production surface, *S*_P_, cannot be assessed and is set to a large value of 100,000, which means that the self-shading at the youth development stage is low.

**Figure 9:**
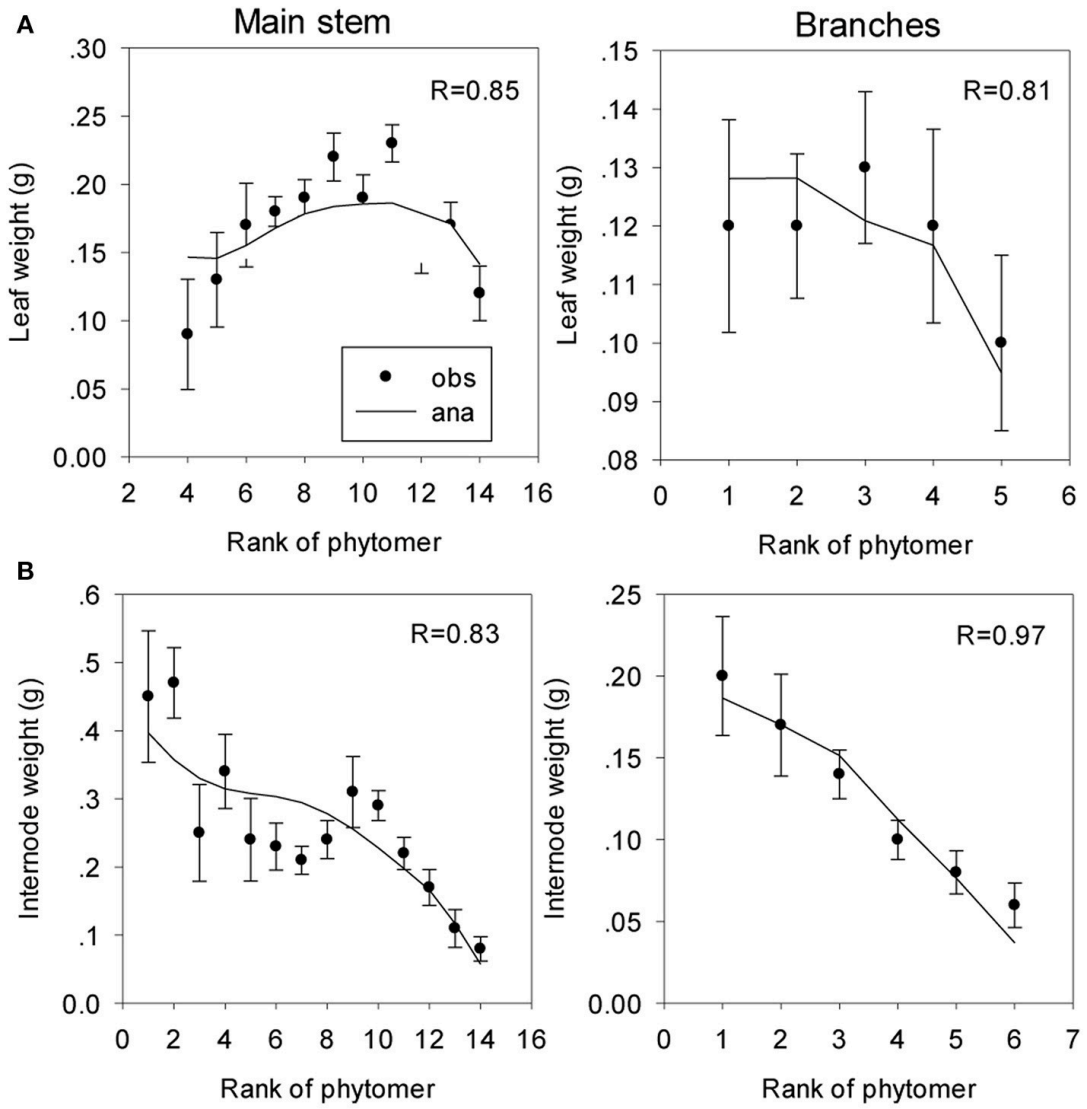
Fitting of organic series of leaves and internodes on the main stem **(A)** and the branches **(B)** of coffee trees. The symbols represent the observed (obs) organic series (circles), and the lines represent the analytical (ana) series. The correlation coefficients (R) between the observed and computational values are shown.

*Parameters from the inverse method* One of the parameters for sink strength evolution (Equation S2) is arbitrarily fixed; another parameter is only computed because the system cannot estimate the two beta law parameters together. The observed and computed organic series are shown on Figure 9. The estimated parameters are listed in Table 2.

**Table 2:** Estimated source and sink parameters of coffee tree.

**Organs**	**Stem sink**	**Branch sink**	**Variation *B_*a*1_***
Blade	1	0.67	1.03
Internodes	0.26	0.19	1.0
Girth growth	0.07	0.07	
*r*	451		
*Q_*o*_*	0.87		

##### Growth-simulation-and-architecture-visualization-of-coffee-trees

**Figure 10:**
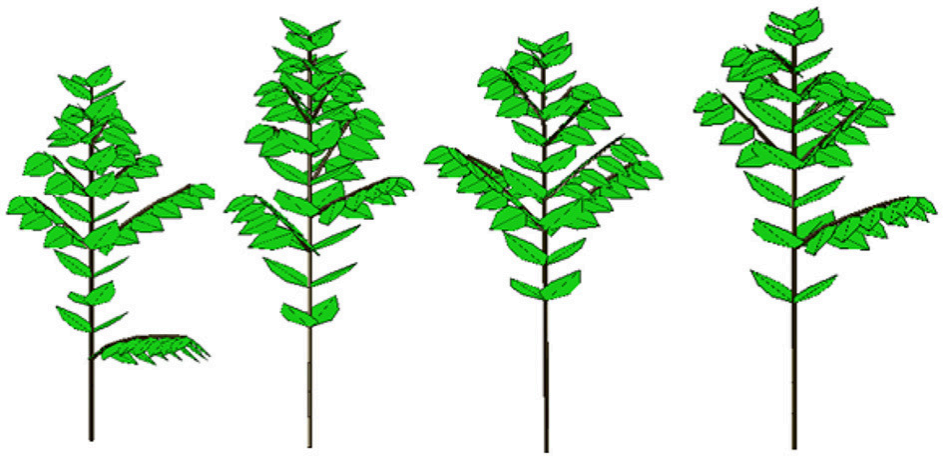
Four 3D stochastic simulations of young *Coffea pseudozanguebariae* coffee trees at DC 16 using Gloups software (Cirad-Amap). Development and growth parameters come from crown and organic series analysis with field measurement data from Ivory Coast by Sélastique Akaffou.

The calibration of the model using the data sets from the coffee tree observations provides the parameters for both development and growth that are necessary and sufficient to simulate plant growth and architecture, as in Figure 10.

### Case of rhythmic development

For the plants with rhythmic development, plant analysis is organized at two levels: GU and phytomer. Crown analysis at the GU level is similar to that of phytomers in the continuous case. Therefore, more attention is paid to the development inside a GU. *In silico* results are given.

**Figure 11:**
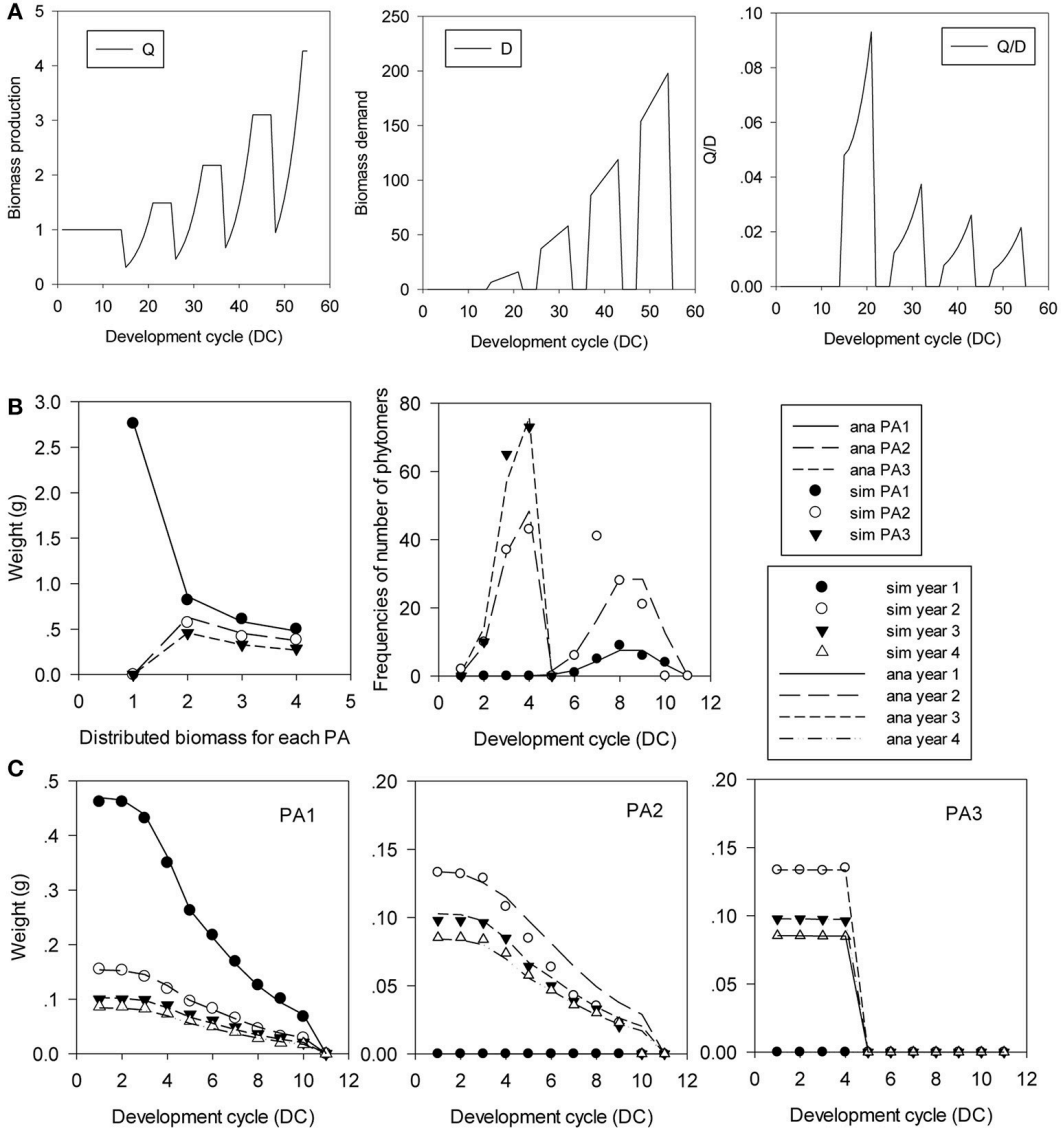
*In silico* analysis and parameter estimation for the Rauh architectural model with rhythmic development. The plant age is 4 GCs. **(A)** Rhythmic growth pattern of biomass production (Q), demand (D) and their ratio (Q/D). **(B)** Biomass profiles of GUs by rank, and the distributions of the number of phytomers per GU. **(C)** Biomass profiles of phytomers in the GU. The simulated (symbols) and analytical (lines) GU counterparts for the 3 PAs and the 4 GCs are in good agreement.

Figure 11 shows this type of tree with 3 PAs. The development of the preformed part is completed in 4 DCs (*T*_1_ = 4), and the neoformed part is distributed according to a positive binomial distribution of *n* = 6 and *b*_1_ = 0.8. Meristem functioning follows a *Bernoulli* process *b* = 0.8. The probabilities of transition to neoformation are *d* = 1, 0.5, and 0 for PA 1, PA 2, and PA 3, respectively. Phytomers are functioning on 10 DCs, and the duration of the 11-DC period is completed by a terminal pause. Organ sinks are equal and constant during the growth period. The simulation in Figure 11 shows the rhythmic patterns.

**Figure 12:**
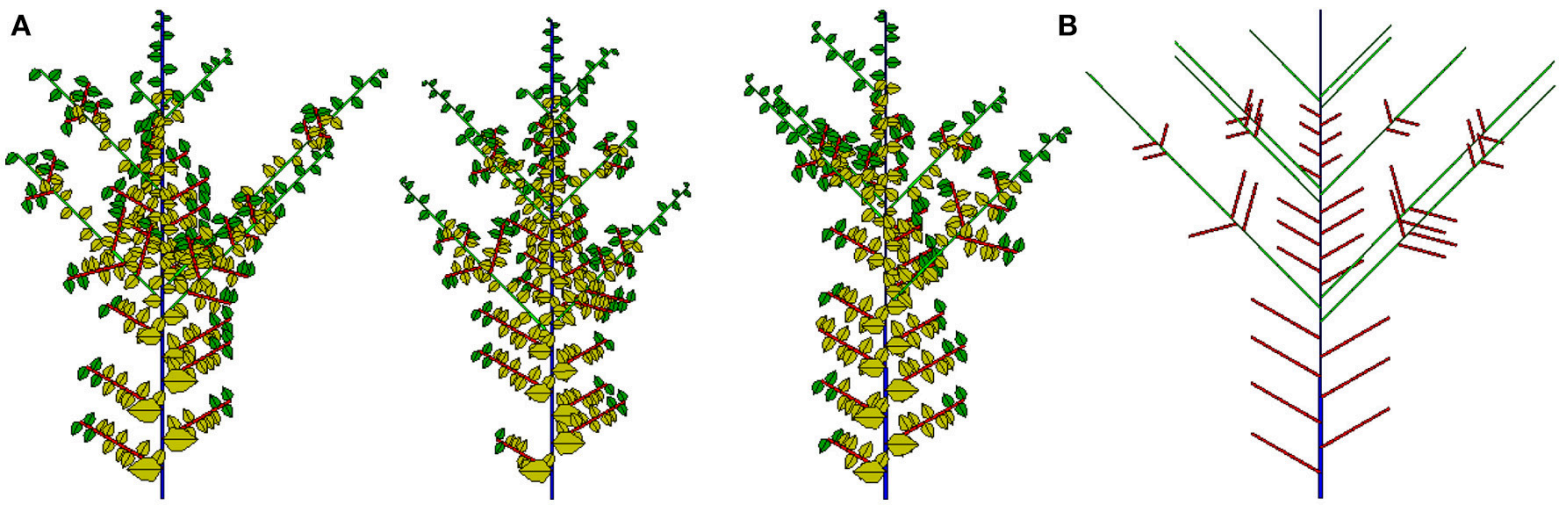
Three stochastic simulations **(A)** and associated potential structure **(B)** for the Rauh architectural model with preformation and neoformation.

The potential structure and three 2D simulated samples are shown in Figure 12. Compared to previous work, an important achievement here is that the analytical outputs of all simulated plants can be evaluated. The most recent application of this work is in the teak tree (Tondjo et al., 2018). Other applications are ongoing.

## Discussion

### The value of organic series

In this work, we have presented how to obtain the analytical organic series for continuous and rhythmic development. In the context of FSPMs, it has previously been difficult to define an average plant because of the variations in branching structures, so to mathematically solve the source and sink functions, one needs both data that are closely related to these functions (the size of organs along stems) and corresponding model outputs that are comparable to the measured data. According to the assumptions of the developmental model, the statistical organic series meet this requirement and thus provide a way to observe and understand plant populations.

The procedure of computing analytical organic series seems complicated, but once it is achieved using software, the series can be much more efficiently obtained from Monte-Carlo simulation. The concept of potential structure adds a new dimension of understanding plants, which can be linked to studies of plant potentials (Kang et al., 2016). For very complex plants for which the analytical output is not available, massive model simulation runs can provide approximate results for comparison with the measured data and to inversely estimate the parameters; this is possible through the rapid advances in computer sciences.

### The importance of the *in silico* exercise

Because of the complexity of both real plants and the FSPM itself, it is risky to fit real data without confidence in the software that is implementing the simulation, analytical computation and parameter estimation of the model. *In silico* plant samples separate the complexity of reality from the model itself, which is an important intermediate step toward the final parameter estimation. Benefiting from the concept of botanical architectural model, in this step, templates of parameter files can be created for plants of different types to test the validity of the model. Further application of the models to real plants can then be decomposed to the choice of the corresponding architectural model and the focus on the specific features of this plant in the template parameter file. This application is performed for the case of the Coffee tree. Which typically belongs to the Roux architectural model. This strategy provides a way to decrease the complexity of modeling work on real plants.

Practically speaking, *in silico* experiments prepare *in vivo* applications for real plants. The work of addressing real plants can begin by replacing simulated target data with observed data from real plants. This eases plant measurement since clear guidance can be given based on *in silico* experiments, including what is to be observed and how to preprocess the observed data. The parameter estimation method decides the method of observing the plant. For the preliminary experimental data, one can set initial parameters that are estimated directly to simulate plants samples that are similar to real ones. Based on this, the target data format can be written by performing virtual sampling of the simulation, which is helpful for organizing real data. Inverse estimation of hidden parameters is performed only in the end, when an approximately plausible parameter file is prepared. Therefore, parameterization is achieved iteratively by running the model and comparing the real and virtual plants. A first trial of the method *in vivo* on a species of coffee tree shows that the GreenLab model can correctly calibrate the development and growth parameters of real plants with stochastic architectures.

### The benefits to plant sampling

As experienced by numerous researchers in the FSPM domain, measuring branching plant structures, such as measuring all the weights and sizes of individual organs at several stages in a wheat, can be very tedious. Sometimes even the measured data cannot be fully used. In this work, the calibration process is performed sequentially: first, the bud probabilities linked to the meristem functioning of each type of axis are estimated to determine plant development, and then the source-sink parameters are estimated using organic series. From a modeling point of view, it is difficult to determine whether the cause is stochastic development or the internal source-sink ratio of a plant, and estimating developmental parameters and then solving the source-sink function provides a way to understand their internal link, such as the cause of fruit set (Ma et al., 2011). Accordingly, records of topological structures and the collection of organ weights can be performed separately on different plants and even by different people. The sampling complexity is therefore reduced. This result could enable further simulations that consider the interaction between plant growth and development (Mathieu et al., 2009).

The organic series contains the history of biomass production and partitioning in a statistical way and provides a simplified means of describing plant growth for parameter estimation. There is no further obligation to fully record the detailed architecture of sample plants that are later destructively measured, as in AMAPMod (Godin et al., 1997). To obtain the organic series, one can sample approximately dozens of (depending on the variation in plant structure) stems and branches from different plants, as shown in Figure 6. This proposed approach is versatile as it does not rely on the complexity of the plant architectural model or its specific stochastic expression. This potentially broadens the application of the model to numerous plants with complex architectures.

### The range of application

Although the results of this approach are interesting, it is not feasible for all plants. For example, in orchard trees such as peach or the kiwi vine, plant development is seriously disrupted by routine pruning or local gravity effects: depending on the condition of the fruits, the bending of branches modifies branching patterns and causes meristem abortions. The notion of a common biomass pool is obviously unsuitable for such plants. It therefore seems very difficult to build a mathematical growth model for the whole plant, and thus a full simulation-oriented modeling scheme appears to be a more suitable way to begin studying the growth of such complex systems. In FSPMs such as L-Peach (Lopez et al., 2008) or L-Kiwi (Cieslak et al., 2011), the models are expressed directly in software that is developed according to certain rules, but the equations that describe the model behavior are missing, and there is no clear strategy for statistical analysis or calibration of the model inversely. Parameters are empirically set according to the literature or miscellaneous experiments and are not the result of model calibration on a single observed plant. These FSPMs provide promising insights into plant behavior through simulation experiments but do not allow the parameters of an observed plant to be assessed.

The GreenLab model describes plant growth and development according to a set of recurrent equations and adapts to most of the 23 architectural models of Hallé et al. (1978). Plants with these architectural models exhibit regular development in their young stages, during which global concepts such as PA and organic series are applicable. Since most crops and the crown part of many trees are intact, the presented approach is relevant to field crops or even trees with regular shapes, such as poplar, pine or conifers. The number of hidden parameters is small because the goal is limited to computing the biomass production and partitioning during growth using integrated parameters. These features make this approach interesting for phenotyping and plant analysis.

### Future work

Applications on other real plants such as spilanthes (Vavitsara et al., 2017), soybean and maple trees have begun and provided the first interesting source and sink parameter estimation results. The Roux and Rauh architectural models presented in this work have been used with many plants and have already broadened applications. Although more effort is needed to explore other architectural models, it is very interesting and challenging to analyze real plants. While the model seems complex, real plants show different types of complexity; for example, the bud probabilities are not necessary stable because of growth gradients, and there could be delays in development and growth (such as in inflorescence). To address such variable cases, dedicated software development is a key issue.

## Conclusion

We have proposed a methodological framework for FSPM parameter estimation for stochastically ramified plants. Focus is given to the estimation of functional parameters inversely by fitting organic series. The analytical mean organic series can be computed based on the potential structure and thus provides an efficient way to measure stochastic plants at the organ level. As a result, an approach similar to that of deterministic plant analysis frameworks can be applied to stochastic plants. *In silico* experiments show that the analytical results converge to the experimental values obtained from sets of Monte-Carlo simulations. Fitting tests through virtual samplings performed on both types of plants prove the feasibility of inverse estimation. The framework is also tested on a real plant (coffee trees) for the case of continuous development. This approach is applicable for plants with continuous and rhythmic development because the same computational methods are used. By sequential calibration of a developmental model and functional model, this method eases the sampling of stochastic branching plant structures, which could otherwise be a burden. The current work breaks a bottleneck in the application of FSPMs to species whose structures show strong variability and can open FSPMs to wider applications.

## Data availability

The software code and datasets for this study can be found at [FIGSHARE] [https://doi.org/10.6084/m9.figshare.6984254].

## Author contributions

MK and XW prepared and revised the manuscript. JH and MJ programmed the computational experiments. PdR conceived the research and proposed the methodology. SA conducted the coffee tree experiment.

### Conflict of interest statement

The authors declare that the research was conducted in the absence of any commercial or financial relationships that could be construed as a potential conflict of interest.
