# Supplementary material for: Estimating Sink Parameters of Stochastic Functional-Structural Plant Models Using Organic Series-Continuous and Rhythmic Development
Source: Front Plant Sci. 2018 Nov 30;9:1688. doi: 10.3389/fpls.2018.01688 (PMC6284058; doi:10.3389/fpls.2018.01688)
Supplement: Supplementary file 1 [file Presentation_1.pdf]

Supplementary Material

Identifying Sink Parameters of Stochastic Functional-Structural Plant Models using Organic Series - Continuous and Rhythmic Development

Mengzhen KANG, Jing HUA, Xiujuan WANG*, Philippe de REFFYE, Marc JAEGER, Akaffou SÉLASTIQUE

*** Correspondence:** Xiujuan Wang: xiujuan.wang@ia.ac.cn

Supplement A: meristem probabilities

Development probability b_p and rhythm ratio w_p

For each DC under the notion of the development cycle, the event during which a meristem produces a phytomer is supposed to take place with a *development probability* b_p, generating a typical *Bernoulli* process, where p is the physiological age of the corresponding axis. At a given DC, a failed *Bernoulli* event leads to a void entity in the time series, meaning that no phytomer is generated (de Reffye et al., 1988). The result is an alternation of random series of realized (1) and void (0) entities (Figure S1). According to the above definition, at a given chronological age n without mortality, the number of phytomers in an axis shows a binomial distribution (n, b).

In plants, different development speeds can be observed between the axes of different branching orders. The ratio between development speeds is called the *rhythm ratio* w (w<1), taking the highest development speed as 1. In this discrete organogenesis model, development is simulated by a periodic series of real and void entities that describes the functioning of the meristem. For example, w=0.75 corresponds to the series 1110 1110 1110 (Figure S1).

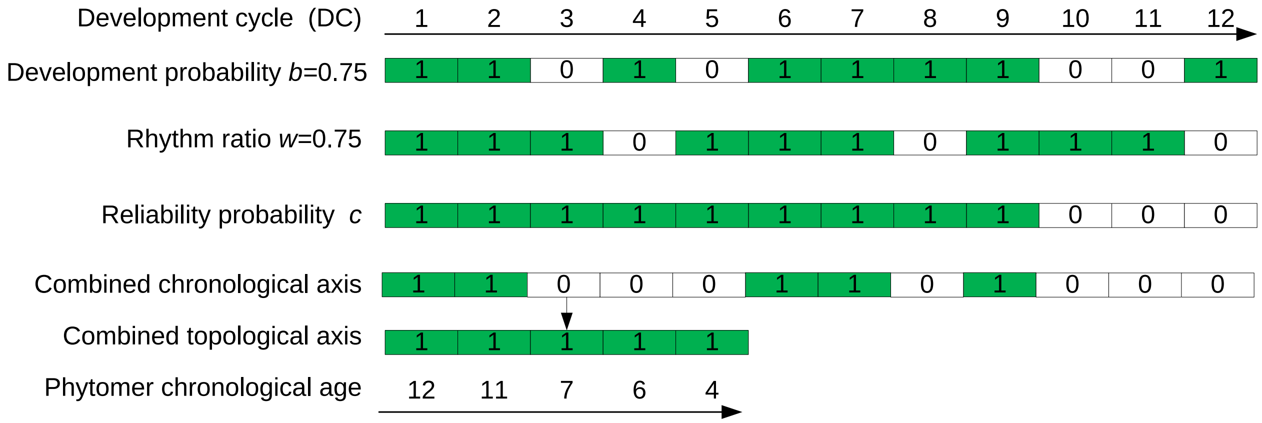

FIGURE S1 Illustration of the effects of development probability, rhythm ratio and reliability probability in the organogenesis model of an axis.

Reliability probability c_p

An axis may end its development for many reasons (*e.g.*, insufficient nutrition or light, insect attack), so at each DC, a *reliability probability* of a meristem is defined, denoted c_p. Once the meristem dies at a given DC, no further phytomers will appear in future DCs, and void entities fill the entire axis from the current phytomer to the tip in the time series (Figure S1). If b=1 and only the reliability probability c takes effect, the number of phytomers follows a geometrical distribution. The combination of both leads to a compound law, mixing dead axes with still-living axes.

The final simulation result and axis is a scalar product between the above three series. The combination leads to a stochastic axis where the chronological age of each phytomer no longer corresponds with its position on the axis (Figure S1), which is the case of real plants.

Branching probability a_p

Considering branching structure, it is supposed that there are a potential number of axillary branches on each phytomer, denoted m (typically being one or two). We consider that a ramification may occur with a *branching probability*, denoted a_p. Accordingly, the probability that the axillary bud never develops a branch is 1-a_p.

Supplement B: the dual-scale automaton (after Kang et al., 2008)

Parallel development of all terminal meristems results in a branching structure. Figure S2 gives an example with the Rauh model, in which each main-stem phytomer can potentially bear one branch. The potential number of phytomers that can bear branches is denoted u_{pq}, with p being the PA of the bearing axis and q being the PA of branches. In this example, u_{12}=u_{13}=1. The mean and the variance in the total number of phytomers in the full branching structure can be obtained through generating functions (Kang et al., 2008).

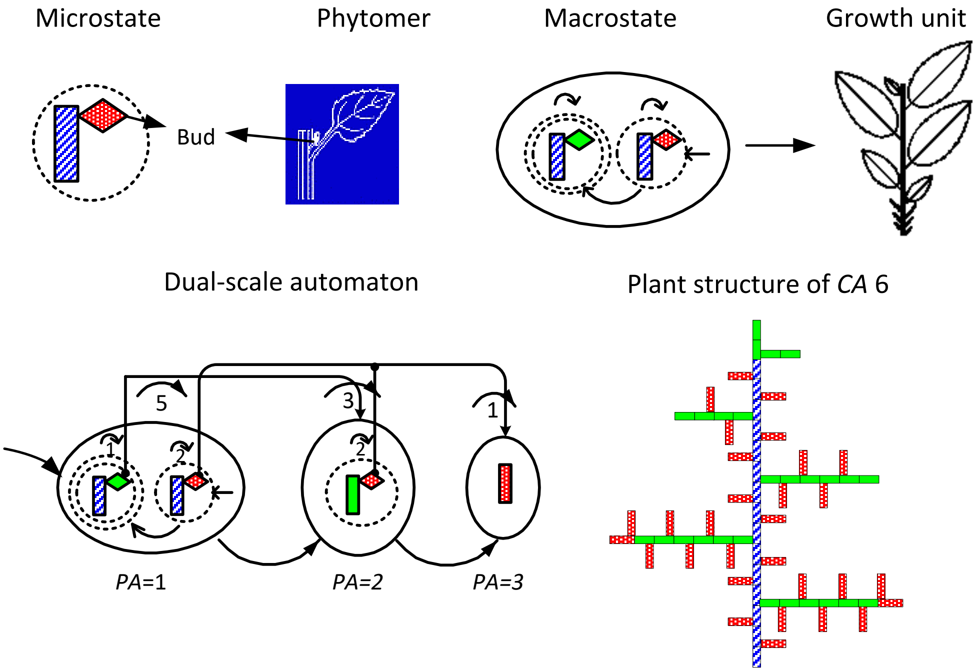

FIGURE S2 Illustration of the dual-scale botanical automaton generating a topological structure of a Rauh architectural model (deterministic/potential development).

Supplement C: modeling stochastic growth

Biomass production

An equation similar to the widely used photosynthesis crop model is used in GreenLab. In the case of a plant with stochastic development, we have the biomass production for a random sample plant *s* in DC *n* in (1):

$$Q^s(n) = LUE \cdot PAR(n) \cdot S_{\mathrm{P}} \cdot \left[1 - \exp\left(-k \cdot \frac{LA^s(n)}{S_{\mathrm{P}}}\right)\right] \qquad \text{(S1)}$$

where *s* is the identification of a stochastic plant sample, being an integer value between 1 and sample size; $Q^s(n)$ is the biomass increment of plant *s* at the *n*-th DC (g plant^{-1} DC^{-1}); *LUE* is the light use efficiency expressed in g·MJ^{-1}; *PAR* is the photosynthetically active radiation above the canopy; *k* is a cultural coefficient issued from leaf orientation; S_{P} is the production surface, which is a hidden parameter, of the root of (1); $LA^s(n)$ is the total plant leaf area of plant *s* at the *n*-th DC. Note that $LA^s(n)$ is a stochastic variable that differs from one plant to another.

Sink functions

In the GreenLab model, the sink value of an individual organ at the *i*-th cycle of its expansion is defined as the product of its sink strength and its sink variation function:

$$P_o^p(i) = p_o^p \cdot F_o\left(\frac{i}{t_o}\right) \qquad \text{(S2)}$$

where p_o^p is the dimensionless relative sink strength of the organ *o* (leaf, internode and fruit) of physiological age *p*; the sink strength of a blade of physiological age 1 (main stem) is set to 1 as a reference; t_o is the duration of the expansion of organ *o,* assumed to be the same for a given physiological age; F_o is an empirical normalized function describing the evolution of sink strength, modeled here by a Beta law (bell shaped) whose parameters are estimated by model inversion (Guo et al., 2006). Sink function is regarded as the same for both the deterministic or stochastic model.

Plant demand

The plant demand at cycle *n*, *i.e.*, its overall biomass request, is the sum of all the active sinks in the growing structure.

$$D^s(n) = \sum_{o.p}\left(\sum_{i=1}^{n} N_o^{p,s}(n-i+1) \cdot P_o^p(i)\right) \qquad \text{(S3)}$$

The plant demand is a convolution on all physiological ages and organ types. $N_o^{p,s}(n\text{-}i+1)$ is the number of organs *o* at cycle *n* resulting from the stochastic organogenesis model, with *i* expansion cycles, born at cycle *n*-*i*+1. Another component of plant demand is that of secondary growth (Kang et al., 2012), which is omitted here for clarity.

Biomass allocation

At each DC n, the incoming biomass, $\Delta q_o^{p,s}(i, n)$, allocated to organ o of age i is proportional to its sink value, $P_o^p(i)$, and the ratio between the plant production $Q^s(n\text{-}1)$ and plant demand $D^s(n)$:

$$\Delta q_o^{p,s}(i,n) = P_o^p(i)\frac{Q^s(n-1)}{D^s(n)} \qquad \text{(S4)}$$

According to this equation, if Q remains the same, fewer created organs will result in smaller plant demand and then larger organs, as can be seen in simulation.

The weight of an organ is then the sum of its increments:

$$q_o^{p,s}(i,n)_{ch} = \sum_{j=1}^{i} P_o^p(j)\cdot\frac{Q^s(n-i+j-1)}{D^s(n-i+j)} \qquad \text{(S5)}$$

Simulated chronological series

Considering organs of different ages, equation (S5) can be written for age n in a matrix form:

$$\begin{bmatrix} q_o^{p,s}(1,n) \\ q_o^{p,s}(2,n) \\ \vdots \\ q_o^{p,s}(n,n) \end{bmatrix} = \begin{bmatrix} P_p^o(1) & 0 & \cdots & 0 \\ P_p^o(2) & P_p^o(1) & \cdots & 0 \\ \vdots & \vdots & \ddots & \vdots \\ P_p^o(n) & P_p^o(n-1) & \cdots & P_p^o(1) \end{bmatrix} \cdot \begin{bmatrix} \frac{Q_{n-1}^s}{D_n^S} \\ \vdots \\ \frac{Q_1^s}{D_2^S} \\ \frac{Q_0}{D_1^S} \end{bmatrix} \qquad \text{(S6)}$$

where $[q_o^{p,s}(*,n)]$ is an n×1 matrix standing for a chronological organic series in an axis of physiological age p;

$\begin{bmatrix} P_p^o(1) & \cdots & 0 \\ \cdots & \cdots & 0 \\ P_p^o(n) & \cdots & P_p^o(1) \end{bmatrix}$ is a triangular matrix containing the sink evolution of the organs according to their chronological ages; and $\left[\frac{Q_{n-1}^s}{D_n^S}\right]$ is an n×1 matrix representing the evolution of the supply to demand ratio along the n cycles. The organic series $[q_o^{p,s}(*,n)]$ contains the memory of the growth of stochastic sample plant s. From multiple stochastic chronological organic series (indexed with ch), one can obtain the average of all organic series $\overline{q_o^p(*,n)_{ch}}$. Choosing multiple growth stages, it is possible to determine the evolution of the organ weights along the ranks.

Leaf area

Organ shape dimension can be retrieved from allometric rules. If the specific leaf weight is e, and the leaf weight is $q_p^{a,s}(i,n)$, then its surface is:

$$S_p(i,n) = \frac{q_p^{a,s}(i,n)}{e} \tag{S7}$$

This enables the individual plant leaf area, LA^s(n), to be computed:

$$LA^s(n) = \frac{1}{e}\sum_p \sum_{i=1}^{min(n,t_a)} N_p^{a,s}(n-i+1)\cdot q_p^{a,s}(i,n) \tag{S8}$$

where t_a is the functioning time of a leaf; $N_p^{a,s}(n-i+1)$ is the number of functioning leaves of chronological age i; and $q_p^{a,s}(i,n)$ is the individual leaf weight.

Recursive form of biomass production

Replacing $q_p^{a,s}(i,n)$ with its value in (S5) finally gives the incoming biomass into the plant at cycle n:

$$Q^s(n) = LUE\cdot PAR(n)\cdot S_{\mathrm{P}}\cdot\left(1-\exp(-\frac{k}{e\cdot S_{\mathrm{P}}}\sum_p\sum_{i=1}^{min(n,ta)} N_p^{a,s}(n-i+1)\sum_{j=1}^{i}\frac{P_a^p(j)Q^s(n-i+j-1)}{D^s(n-i+j)})\right)$$
(S9)

The system begins at n=1 from Q_0 provided by the seed. The analytical biomass production $Q^\theta(n)$ shares the same form.

Supplement D: estimating parameters for development - plant crown analysis (after Diao et al., 2012)

The upper part of a plant, or plant crown, is chosen for sampling and provides the data source, as this part is usually the most intact and thus contains the best information about meristem activities. The crown analysis retrieves the development parameters (a, b and c) from the plant architecture measurements (Diao et al., 2012). This approach has been used for numerous species such as coffee (de Reffye et al., 1990), bamboo, cotton, and eucalyptus (Diao et al., 2012). The principles are restated here.

Estimating development parameters b_1 and b_2 and rhythm ratio w

Take T crowns for analysis with each defined by an axis and the set of first-order branches on it. Note that only living branch axes are chosen for observation. For each axis, the position (phytomer rank) of the branch from the top (noted as K) is recorded, and the number of phytomers in the branch axis (noted as $X_{s,K}$, $s\epsilon[1, T]$ being the identification of the sampled plant crown). The aim is to estimate the development probability for the axis (b_1) and the branch axis (b_2) as well as the rhythm ratio between them (w) (that of the main axis is set to 1 as a reference).

These probabilities can be estimated from the mean and variance of the number of phytomers in branches located at different positions. In case there are branches of higher order, this analysis can be applied to a pair of orders two and three and so on to obtain the corresponding parameters (Diao et al., 2012).

Suppose there are T crowns to be analyzed, each defined by a stem and the set of branches on it. At rank K from the top of the stem, there is a global mean and a global variance for the number of phytomers of the corresponding branches:

The observed global mean is:

$$\overline{X_K} = \frac{\sum_{s=1}^{T} X_{s,K}}{T} \tag{S10}$$

While the analytical value is:

$$X_k = w\frac{K}{b_1}b_2 \tag{S11}$$

The observed global variance is:

$$\overline{V_k} = \sum_{s=1}^{T}\frac{(X_{s,k} - \overline{X_K})^2}{T-1} \tag{S11}$$

The analytical global variance is:

$$V_K = w\frac{K}{b_1}b_2(1-b_2) + (w\cdot b_2)^2\frac{K(1-b_1)}{{b_1}^2} \tag{S12}$$

The local variance between two branches on the same stem separated by L phytomers is:

$$\overline{v_K}(L) = \frac{\sum_{s=1}^{T}(X_{s,K} - X_{s,K+L})^2}{2T} \tag{S13}$$

The analytical local variance is:

$$v_K(L) = w\cdot b_2(1-b_2)\frac{K+\frac{L}{2}}{b_1} + (w\cdot b_2)^2\frac{L(1-b_1)}{2{b_1}^2} + (w\cdot b_1)^2\frac{L^2}{2{b_2}^2} \tag{S14}$$

Solving the system, we obtain b_1, b_2 and w:

$$1-b_2 = \frac{1}{X_K}\left(v_K - \frac{L}{2}\left(\frac{V_K}{K} + L\left(\frac{X_K}{K}\right)^2\right)\right)$$

$$1-b_1 = \frac{1}{{X_K}^2}\left(K\left(V_K - v_K + \frac{1}{2}\left(\frac{L\cdot X_K}{K}\right)^2\right) + \frac{L\cdot V_K}{2}\right) \tag{S15}$$

$$w = \frac{b_1}{b_2}\cdot\frac{X_K}{K}$$

The computation of b_1, b_2 and w can be done for each selected rank k, and when more k are selected, their average values are more accurate. For two branches belonging to the same whorl, we have L=0, while we have L=1 for two adjacent branches.

Estimating the branching probability *a*

The branching rate at rank K, $a(K)$, is estimated as the ratio between the number of observed branches and the number of available axillary meristems.

Estimating the reliability probability *c*

The abortion rate $Fc_1(K)$ at the phytomer of rank K is the ratio between number of the dead branches and the total number of branches observed. This proportion depends on the probability of bud survival c_i at each DC i from 1 to n. Data assimilation is performed using the mean of target files to be fitted by the model that describe the status of the phytomers on the stem at rank K from the axis tip, *i.e.*, how many phytomers get branches and whether they are alive or dead. Due to the *Bernoulli* process, the average age at rank K is:

$$n = \frac{K}{b_1} \qquad \text{(S16)}$$

Rewriting the rank-governed reliability function $Fc_1(K)$ on the thermal time basis $Fc_2(n)$ using equation, the reliability, c_n, of the meristem, *i.e.*, the probability to survive at cycle n, can be deduced from the following recurrence system:

$$\begin{aligned} c_1 &= 1 - Fc_2(0) \\ c_n &= \frac{1 - Fc_2(n)}{1 - Fc_2(n-1)} \end{aligned} \qquad \text{(S17)}$$

If parameter c is constant, we have:

$$\mathrm{F}_{\mathrm{C}_2}(\mathrm{n}) = 1 - \mathrm{c}^{\mathrm{n}} \qquad \text{(S18)}$$

Generally, Fc_2 is a sigmoid that can be assessed by two parameters, B_a and B_b, in a process of n duration from meristem birth to death.

$$F_{C2}(i) = \frac{\sum_{i=1}^{T} \left(\frac{i}{n}\right)^{B_a} \left(1 - \frac{i}{n}\right)^{B_b}}{\sum_{i=1}^{T} \left(\frac{i}{n}\right)^{B_a} \left(1 - \frac{i}{n}\right)^{B_b}} \qquad \text{(S19)}$$

Supplement E: Example of the experiments of simulation and computation using the software 'Gloups'

The experiments of simulation and computation was performed in the software ‘Gloups’ developed under Windows environment using MATLAB. The main procedures include one for simulation and another for calibration.

Simulation of stochastic development

Monte-Carlo method is applied for the simulation of stochastic development. In Gloups, ‘ZSub_GL5_2S’ is used for the simulation. The growth cycle of plants can be set as needed in ‘ZSub_GL5_2S’.

For Figure 5, run ‘ZSub_GL5_2S’, choose parameter file ‘GLOUP\Target\Fig 5_par.m’, the potential structure (A) can be drawn.

To obtain the simulated chronological organic series for a single stem, the parameter values in Line 4 needs to be set to 1 for chronological and 0 for topological mode, and that in Line178 needs to be set to 0.7 both for the main stem and branches, as follows:

mode_chrono_biomass___msink____use_msk____CuG___crown___Topdown___Botup___onto
1 1 0 0 0 0 0 0 0 2

Rd_(B_Pv_Growth_phyt_Gu_proba_164_166)
0.7 0.7 1 1 1 0 0 0 0 89

For Figure 6, run ‘ZSub_GL5_2S’, choose parameter file ‘GLOUP\Target\Fig 6_par.m’, then the virtual target data file can be obtained in ‘targ.m’, and the simulated plant structures for picking organic series can be obtained. The value of ‘Draw_Rep_Plts’ of Line42 in ‘ZSub_GL5_2S’ can be set to the number needed for the numbers of structures.

Calibration of stochastic development

In Gloups, ‘ZSub_GL5_2S’ is used for the parameter estimation. In calibration, it calls the analytical computation of organic series according to initial parameters values, the result is to be fit with the target data, either from simulation, or from real plants.

5.2.1 Calibration from the virtual target data

For Figure 6, as we have obtained the virtual target data ‘targ.m’ renamed as ‘Fig6_tar.m’, we can estimate the parameters using ‘ZSub_GL5_2A’. Run ‘ZSub_GL5_2A’, choose the parameter file ‘Fig 6_par.m’ and ‘Fig 6_tar.m’, the parameter values can be obtained, and the estimated weights of each organ according to their positions, the source-sink ratio, etc., will be given. Generally speaking, the source-sink parameters can be estimated simultaneously or separately. In the parameter files, we can choose which parameters we want to compute.

5.2.2 Calibration from the simulated target data

For Figure 9, as we have obtained the measured data (weights of blade and internode, number of phytomers on the main stem and branches), first, we compute the probabilities of a, b and w with the crown analysis; Second, the values of these probabilities can be set in the parameter file ‘Fig9_par.m’; Third, run ‘ZSub_GL5_2S’, choose parameter file ‘GLOUP\Target\Fig 9_par.m’, thus, the virtual

target data file can be obtained in 'targ.m', then replaced the target data by the measured data, named as 'Fig9_tar.m'; Finally, we can estimate the parameters with the method mentioned above.

Supplement F: list of abbreviations and parameters

**Abbreviation**	**Description**	**Source**
CA	Chronological age	Page 3, Section Continuous-development
ch	Index of chronological organic series	Section Analytical chronological series; Equations 4 and 6
DC	Development cycle	Section Continuous-development
GC	Growth cycle	Section Rhythmic-development
GU	Growth unit	Section Rhythmic-development
LUE	Light use efficiency	Section Parameter estimation test in the Roux architectural model; Equation S1
PA	Physiological age	Section Modeling the Development of Branching Structure
PAR	Photosynthetically active radiation	Supplement C; Eqation S1
tp	Index of topological series	Sec Analytical topological series; Eqations 6, 7 and 8
**Parameter**	**Description**	**Source**
a	Branching probability	Page 4, Section Modeling the development of branching structure
b	Development probability	Page 3, Section Continuous-development; Equation 5
b_i	Probability of existence of the phytomer	Page 8, Equation 12
c	Reliability probability	Page 4, Section Continuous-development
d	Probability of passing from preformation to neoformation	Page 7, Section DC-level; Equation 10
$D^{\theta}(n)$	Plant demand	Page 6, Section Analytical demand; Equation 1
e	Specific leaf weight	Page 9, Section Parameter-estimation-test-in-the-Roux-architectural-model; Supplement C; Equation S7
F	Distribution of the number of phytomers of the GCs with pre- and neoformation	Page 7, Section DC-level; Equation 10
F_o	Empirical normalized function describing the evolution of sink strength	Page 9, Case of Continuous Development; Supplement C; Equation S2
G	Distribution of the number of phytomers per GU	Page 7, Section DC-level; Equation 10
K	Position of the branch from the top	Page 7, Section Analytical Topological Series; Equation 5
k	Cultural coefficient issued from leaf orientation	Supplement C; Equation S1

$LA^s(n)$	Total plant leaf area of plant *s* at the *n*-th CD	Supplement C; Equation S1
max*p*	Highest PA of the plant	Page 4, Section Modeling the development of branching structure
n	Plant/axis age	Page 6, Section Analytical demand; Equation 1
$N_o^{p,s}(n)$	Number of organs in a random plant sample *s* of type *o* and physiological age *p* appeareding at plant age *n*	Supplement C; Equation S3
o	Organ type	Page 6, Section Analytical demand; Equation 1
P	Distribution of the number of phytomers of the GCs for the pre-formation part	Page8, Section DC-level; Equation 9
p	Physiological age	Page 4, Section Modeling the development of branching structure; Equation 1
p_o^p	Dimensionless relative sink strength of the organ *o* (leaf, internode and fruit) of physiological age *p*	Supplement C; Equation S2
pu_{ij}	Parameter of the multinomial law	Page 4, Section Modeling the development of branching structure
Q_0	Seed weight	Page 13, Table 2; Supplement C
$Q^s(n)$	Biomass production at cycle *n* for a randomly sampled plant *s*	Supplement C; Equation S3
$Q^\theta(n)$	Analytical biomass production at cycle *n*	Page 6, Section Analytical demand
s	ID of stochastic plant sample	Page 6, Section Analytical demand; Equation 1
S_P	Production surface	Supplement C; Equation S1
t	Number of organ types	Page 5, Section Organic Series
T	Number of crowns	Page 7, Section Analytical Topological Series; Equation 7
N_1	Duration of creating phytomers in preformation part, for rhythmic development	Page 4, Section Rhythmic-development
N_2	Duration of creating phytomers in neoformation part, for rhythmic development	Page 4, Section Rhythmic-development
t_a	Functioning time of a leaf	Supplement C; Equation S8
N_d	Duration (in DC) of development, sum of N_1 and N_2, for rhythmic development.	Page 8, Section DC-level
t_o	Duration of the expansion of organ *o*	Supplement C; Equation S2

N_u	Duration (in DC) of a GU, for rhythmic development.	Page 4, Section Rhythmic-development
w	Rhythm ratio	Supplement A
	Analytical average	Page 6, Section Analytical demand; Equation 1

References

Barthélémy, D., and Caraglio, Y. (2007). Plant Architecture: A Dynamic, Multilevel and Comprehensive Approach to Plant Form, Structure and Ontogeny. *Annals of Botany* 99(3)**,** 375-407.

de Reffye, P., Snoeck, J., and Jaeger, M. (1990). "Modelling and simulation of the growth and architecture of the coffee tree", in: *13th International scientific colloquium on coffee*), 21-25.

Diao, J., De Reffye, P., Lei, X., Guo, H., and Letort, V. (2012). Simulation of the topological development of young eucalyptus using a stochastic model and sampling measurement strategy. *Computers and Electronics in Agriculture* 80**,** 105-114.

Guo, Y., Ma, Y., Zhan, Z., Li, B., Dingkuhn, M., Luquet, D., et al. (2006). Parameter Optimization and Field Validation of the Functional-Structural Model GREENLAB for Maize. *Annals of Botany* 97(2)**,** 217-230.

Kang, M.Z., Cournede, P.H., de Reffye, P., Auclair, D., and Hu, B.G. (2008). Analytical study of a stochastic plant growth model: Application to the GreenLab model. *Mathematics and Computers in Simulation* 78(1)**,** 57-75.
